# Admissibility of simultaneous prediction for actual and average values in finite population

**DOI:** 10.1186/s13660-018-1707-x

**Published:** 2018-05-16

**Authors:** Chao Bai, Haiqi Li

**Affiliations:** 1 School of Finance and Statistics, Hunan University, Changsha, China; 2 College of Science, Central South University of Forestry and Technology, Changsha, China

**Keywords:** 62C15, 62M20, 62J12, 15A39, Admissibility, Simultaneous prediction, Quadratic loss function, Generalized linear regression model

## Abstract

This paper studies the admissibility of simultaneous prediction of actual and average values of the regressand in the generalized linear regression model under the quadratic loss function. Necessary and sufficient conditions are derived for the simultaneous prediction to be admissible in classes of homogeneous and nonhomogeneous linear predictors, respectively.

## Introduction

We begin this paper with some notation first. For an $m\times n$ matrix ***A***, the symbols $\mathscr{M} (\boldsymbol{A})$, $\boldsymbol{A}'$, $\boldsymbol{A}^{-}$ and $\boldsymbol{A}^{+}$ denote the column space, the transpose, the generalized inverse and Moore–Penrose inverse of ***A***, respectively. For squared matrices ***B*** and ***C***, $\boldsymbol{B}\geq \boldsymbol{C}$ means that $\boldsymbol{B}-\boldsymbol{C}$ is a symmetric nonnegative definite matrix and $\boldsymbol{B}-\boldsymbol{C}\geq\mathbf{0}$. Let $\operatorname{rk}(\boldsymbol{A})$ be the rank of ***A*** and $\operatorname{tr}(\boldsymbol{A})$ be the trace of ***A*** when ***A*** is a squared matrix. ***I*** is an identity matrix with an appropriate order and $\mathbb{R}^{n}$ denotes the *n* dimensional vector set.

Consider the following generalized linear regression model:

1 $$ \boldsymbol{y}=\boldsymbol{X}\boldsymbol{\beta}+\boldsymbol{\varepsilon},\qquad \text{E}(\boldsymbol{\varepsilon})=\textbf {0},\qquad \operatorname{Cov}( \boldsymbol{\varepsilon})=\boldsymbol{\Sigma}, $$

where ***y*** is the $n\times1$ observable vector of regressand, ***X*** is the $n\times p$ observable matrix of regressor, ***β*** is a $p\times1$ unknown vector of regression coefficient, ***ε*** is the $n\times1$ vector of disturbances and $\boldsymbol{\Sigma}\geq\mathbf{0}$. Suppose $\operatorname{rk}(\boldsymbol{X})\leq p$.

Given the matrix of regressor $\boldsymbol{X}_{0}$ (which is correlated with new observations), the relationship between the unobservable random vector $\boldsymbol{y}_{0}$ and $\boldsymbol{X}_{0}$ is

2 $$ \boldsymbol{y}_{0}=\boldsymbol{X}_{0}\boldsymbol{\beta}+\boldsymbol{\varepsilon}_{0},\qquad \text{E}(\boldsymbol{\varepsilon}_{0})=\mathbf{0},\qquad \operatorname{Cov}(\boldsymbol{\varepsilon}_{0})=\boldsymbol{\Sigma}_{0}, $$

where ***y*** is the $m\times1$ vector of the regressand to be predicted, $\boldsymbol{X}_{0}$ is the $m\times p$ matrix of prediction regressor, ***β*** is the same as that in model (1), $\boldsymbol{\varepsilon}_{0}$ is the $m\times1$ vector of prediction disturbances and $\boldsymbol{\Sigma}_{0}\geq\mathbf{0}$. Assume ***y*** and $\boldsymbol{y}_{0}$ are correlated and $\operatorname{Cov}(\boldsymbol{\varepsilon}_{0},\boldsymbol{\varepsilon}')=\boldsymbol{V}$. Suppose the finite population is composed of ***y*** and $\boldsymbol{y}_{0}$. Thus, combining models of (1) and (2), we have

3 $$ \boldsymbol{y}_{T}=\boldsymbol{X}_{T}\boldsymbol{\beta}+\boldsymbol{\varepsilon}_{T}, $$

where

$$\begin{array}{c} y_{T}=\left(\begin{array}{c} y\\ y_{0} \end{array}\right),\;X_{T}=\left(\begin{array}{c} X\\ X_{0} \end{array}\right),\;\varepsilon_{T}=\left(\begin{array}{c} \varepsilon \\ \varepsilon_{0} \end{array}\right),\\ \text{E}\varepsilon_{T}=0,\;\mathrm{Cov}\varepsilon_{T}=\mathrm{Cov}\left(\begin{array}{c} \varepsilon \\ \varepsilon_{0} \end{array}\right)=\left(\begin{array}{cc} \Sigma & V^{\prime }\\ V & \Sigma_{0} \end{array}\right)=\Sigma_{T}. \end{array}$$

For the prediction in model (3), [1] obtained the best linear unbiased predictor (BLUP) of $\boldsymbol{y}_{0}$. [2] considered the optimal Stein-rule prediction. Reference [3] investigated the admissibility of linear predictors with inequality constraints under the quadratic loss function. [4] reviewed the existing theory of minimum mean squared error (MSE) predictors and made an extension based on the principle of equivariance. [5] derived the BLUP and the admissible predictor under the matrix loss function. Under the MSE loss function, the optimal predictor of $\boldsymbol{y}_{0}$ is the conditional expectation $\text{E}(\boldsymbol{y}_{0}|\boldsymbol{X}_{0})=\boldsymbol{X}_{0}\boldsymbol{\beta}$, which relates naturally to the plug-in estimators of ***β***. [6] proposed the simple projection predictor (SPP) of $\boldsymbol{X}_{0}\boldsymbol{\beta}$ by plugging in the best linear unbiased estimator (BLUE) of ***β***. The plug-in approach spawned a large literature for the derivation of combined prediction; see [7, 8], etc.

Generally, predictions are investigated either for $\boldsymbol{y}_{0}$ or for $\text{E}\boldsymbol{y}_{0}$ at a time. However, sometimes in the fields of medicine and economics, people would like to know the actual value of $\boldsymbol{y}_{0}$ and its average value $\text{E}\boldsymbol{y}_{0}$ simultaneously. For example, in the financial markets, some investors may want to know the actual profit while others would be more interested in the average profit. Therefore, in order to meet different requirements, the market manager should acquire both the prediction of the actual profit and the prediction of the average profit at the same time, and can assign different weights to each prediction to provide a more comprehensive combined prediction of the profit. Under these circumstances, we consider the following target function:

$$\boldsymbol{\delta}=\lambda \boldsymbol{y}_{0}+(1-\lambda) \text{E}\boldsymbol{y}_{0}, $$

where ${\lambda}\in[0,1]$ is a non-stochastic scalar representing the preference to the prediction of the actual and average values of the studied variable. A prediction of ***δ*** is the simultaneous prediction of the actual and average values of $\boldsymbol{y}_{0}$. Note that $\boldsymbol{\delta}=\boldsymbol{y}_{0}$ if $\lambda=1$ and $\boldsymbol{\delta}=\text{E}\boldsymbol{y}_{0}$ if $\lambda=0$, which means the studied ***δ*** is more sensitive and inclusive.

Studies on the simultaneous prediction of the actual and average values of the studied variable (namely prediction of ***δ***) have been carried out in the literature from various perspective. The properties of the predictors by plugging in Stein-rule estimators have been concerned by [9] and [10]. [11] investigated the Stein-rule prediction for ***δ*** in linear regression model when the error covariance matrix was positive definite yet unknown. References [12, 13] and [14] considered predictors for ***δ*** in linear regression models with stochastic or non-stochastic linear constraints on the regression coefficients. The issues of simultaneous prediction in measurement error models have been addressed in [15] and [16]. [17] considered a matrix multiple of the classical forecast vector for the simultaneous prediction of and discussed the performance properties.

This paper aims to study the admissibility of simultaneous prediction of actual and average values of the unobserved regressand in finite population under the quadratic loss function. Admissibility is an interesting problem in statistical theory and received much attention. [18, 19] and [20] discussed the admissibility of predictions of $\boldsymbol{y}_{T}$. [21, 22] and [23] studied the admissibility of estimations of ***β***. We discuss the admissible predictors of *δ* in classes of homogeneous and nonhomogeneous linear predictors, respectively. Necessary and sufficient conditions for the simultaneous prediction to be admissible are provided.

The rest of this paper is organized as follows. In Sect. 2, we give some preliminaries. In Sect. 3, we obtain the homogeneous linear admissible simultaneous predictors for the actual and average values of the unobserved regressand. In Sect. 4, we derive the necessary and sufficient conditions for linear simultaneous prediction to be admissible in class of nonhomogeneous linear predictors. Concluding remarks are placed in Sect. 5.

## Preliminaries

Suppose *d* is the predictor of *δ* and denote $\text{R}(d;\beta)$ as the risk of *d* under the quadratic loss function, then for model (3)

$$\text{R}(d;\beta)=\text{E}(d-\delta)'(d-\delta)=\text{E}\bigl[d-{\lambda}y_{0}-(1-{\lambda})X _{0}\beta\bigr]'\bigl[d-{\lambda}y_{0}-(1-{\lambda})X_{0}\beta\bigr]. $$

Denote the classes of homogeneous and nonhomogeneous linear predictors, respectively, by

$$\begin{aligned}& \mathscr{L}\mathscr{H}=\{\boldsymbol{C}\boldsymbol{y}\mid \boldsymbol{C}\mbox{ is an } m \times n \mbox{ matrix}\}, \quad\mbox{and} \\& \mathscr{LN}=\{\boldsymbol{C}\boldsymbol{y}+\boldsymbol{u}\mid C \mbox{ is an } m\times n \mbox{ matrix and } \boldsymbol{u}\mbox{ is an } m\times1 \\& \hphantom{\mathscr{LN}=} \mbox{nonstochastic vector and } \boldsymbol{u}\neq 0\}. \end{aligned}$$

The nonhomogeneous linear predictor is actually an adjustment of the homogeneous linear predictor. We study the admissibility of the prediction of ***δ*** in $\mathscr{L}\mathscr{H}$ and $\mathscr{LN}$. Before the discussion begins, we first present some important preliminaries and basic results.

### Definition 2.1

A predictor ***d*** is said to be admissible for ***δ*** under the quadratic loss function, denoted $\boldsymbol{d}\sim \boldsymbol{\delta}$, iff there exists no other predictor $\boldsymbol{d}^{*}$ such that $\text{R}[\boldsymbol{d}^{*};\boldsymbol{\beta}]\leq \text{R}[\boldsymbol{d};\boldsymbol{\beta}]$ with strict inequality holding at least at one value of ***β***. ***d*** is called better than $\boldsymbol{d}^{*}$ iff $\text{R}[\boldsymbol{d};\boldsymbol{\beta}]\leq \text{R}[\boldsymbol{d}^{*};\boldsymbol{\beta}]$ with strict inequality holding at least at one value of ***β***.

### Definition 2.2

***d*** is an unbiased predictor of ***δ*** if $\text{E}\boldsymbol{d}=\text{E}\boldsymbol{\delta}$.

### Lemma 2.1

([24])

*If* $\boldsymbol{C}\boldsymbol{y}\sim \boldsymbol{y}_{0}$, *then for*, *any* $l\times m$ *matrix* ***L***, $\boldsymbol{L}\boldsymbol{C}\boldsymbol{y}\sim \boldsymbol{L}\boldsymbol{y}_{0}$.

### Lemma 2.2

([25])

*If the square matrix* ***B*** *is not symmetrical*, *then there exists an orthogonal matrix* ***P*** *such that* $\operatorname{tr}(\boldsymbol{P}\boldsymbol{B})=\operatorname{tr}(\boldsymbol{B})$.

### Lemma 2.3

*Suppose* ***Cy*** *is an arbitrary predictor of* ***δ*** *in model* (3). *Let*

$$\widetilde{\boldsymbol{C}}=\boldsymbol{C}\boldsymbol{X}\bigl(\boldsymbol{X}'\boldsymbol{T}^{+}\boldsymbol{X}\bigr)^{-}\boldsymbol{X}'\boldsymbol{T}^{+}+\lambda \boldsymbol{V}\boldsymbol{T}^{+}\bigl[\boldsymbol{I}-\boldsymbol{X}\bigl(\boldsymbol{X}'\boldsymbol{T}^{+}\boldsymbol{X}\bigr)^{-}\boldsymbol{X}'\boldsymbol{T}^{+}\bigr], $$

*where* $\boldsymbol{T}=\boldsymbol{X}\boldsymbol{X}'+\boldsymbol{\Sigma}$. *Then under the quadratic loss function*

$$\text{R}(\boldsymbol{C}\boldsymbol{y};\boldsymbol{\beta})\geq \text{R}(\widetilde{\boldsymbol{C}}\boldsymbol{y};\boldsymbol{\beta}) $$

*for every* $\boldsymbol{\beta}\in\mathbb{R}^{p}$ *and the equation holds if and only if either of the following two conditions holds*:

1. $\boldsymbol{C}\boldsymbol{\Sigma}=\widetilde{\boldsymbol{C}}\boldsymbol{\Sigma}$;
2. $\mathscr{M}(\boldsymbol{\Sigma} \boldsymbol{C}'-{\lambda} \boldsymbol{V}')\subseteq\mathscr {M}(\boldsymbol{X})$.

### Proof

By direct calculation,

$$\begin{aligned} &\text{R}(\boldsymbol{C}\boldsymbol{y};\boldsymbol{\beta}) \\ &\quad=\text{E}\bigl[\boldsymbol{C}\boldsymbol{y}-{\lambda} \boldsymbol{y}_{0}-(1-{\lambda})\boldsymbol{X}_{0}\beta \bigr]'\bigl[\boldsymbol{C}\boldsymbol{y}-{\lambda} \boldsymbol{y}_{0}-(1-{\lambda})\boldsymbol{X}_{0} \beta\bigr] \\ &\quad=\operatorname{tr}\bigl(\boldsymbol{C}\boldsymbol{\Sigma} \boldsymbol{C}'+\lambda^{2}\boldsymbol{\Sigma}_{0}-2\lambda \boldsymbol{C}\boldsymbol{V}'\bigr)+\boldsymbol{\beta}'(\boldsymbol{C}\boldsymbol{X}- \boldsymbol{X}_{0})'(\boldsymbol{C}\boldsymbol{X}-\boldsymbol{X}_{0})\boldsymbol{\beta}\\ &\quad=\text{E}\bigl\{ {\lambda}(\boldsymbol{C}\boldsymbol{y}-\boldsymbol{y}_{0})'(\boldsymbol{C}\boldsymbol{y}-\boldsymbol{y}_{0})+(1-{\lambda}) (\boldsymbol{C}\boldsymbol{y}-\boldsymbol{X}_{0}\boldsymbol{\beta})'(\boldsymbol{C}\boldsymbol{y}- \boldsymbol{X}_{0}\boldsymbol{\beta})\bigr\} +\bigl(\lambda^{2}-\lambda\bigr)\operatorname{tr} \boldsymbol{\Sigma}_{0} \\ &\quad=\text{E}\bigl\{ {\lambda}(\boldsymbol{C}\boldsymbol{y}-\widetilde{\boldsymbol{C}}\boldsymbol{y}+\widetilde{\boldsymbol{C}}\boldsymbol{y}-\boldsymbol{y}_{0})'(\boldsymbol{C}\boldsymbol{y}- \widetilde{\boldsymbol{C}}\boldsymbol{y}+\widetilde{\boldsymbol{C}}\boldsymbol{y}-\boldsymbol{y}_{0}) \\ &\qquad{}+(1-{\lambda}) (\boldsymbol{C}\boldsymbol{y}-\widetilde{\boldsymbol{C}}\boldsymbol{y}+\widetilde{\boldsymbol{C}}\boldsymbol{y}-\boldsymbol{X}_{0} \boldsymbol{\beta})'(\boldsymbol{C}\boldsymbol{y}- \widetilde{\boldsymbol{C}}\boldsymbol{y}+\widetilde{\boldsymbol{C}}\boldsymbol{y}-\boldsymbol{X}_{0}\boldsymbol{\beta})\bigr\} +\bigl(\lambda^{2}- \lambda\bigr)\operatorname{tr} \boldsymbol{\Sigma}_{0} \\ &\quad=\text{E}\bigl\{ (\boldsymbol{C}\boldsymbol{y}-\widetilde{\boldsymbol{C}}\boldsymbol{y})'(\boldsymbol{C}\boldsymbol{y}-\widetilde{\boldsymbol{C}}\boldsymbol{y})+ {\lambda}( \widetilde{\boldsymbol{C}}\boldsymbol{y}-\boldsymbol{y}_{0})'(\widetilde{\boldsymbol{C}}\boldsymbol{y}-\boldsymbol{y}_{0}) +(1-{\lambda}) ( \widetilde{\boldsymbol{C}}\boldsymbol{y}-\boldsymbol{X}_{0} \boldsymbol{\beta})'(\widetilde{\boldsymbol{C}}\boldsymbol{y}-\boldsymbol{X}_{0}\boldsymbol{\beta}) \\ &\qquad{}+2{\lambda}(\boldsymbol{C}\boldsymbol{y}-\widetilde{\boldsymbol{C}}\boldsymbol{y})'(\widetilde{\boldsymbol{C}}\boldsymbol{y}-\boldsymbol{y}_{0})+2(1-{\lambda}) (\boldsymbol{C}\boldsymbol{y}- \widetilde{\boldsymbol{C}}\boldsymbol{y})'(\widetilde{\boldsymbol{C}}\boldsymbol{y}-\boldsymbol{X}_{0}\boldsymbol{\beta})\bigr\} +\bigl(\lambda^{2}-\lambda\bigr) \operatorname{tr} \boldsymbol{\Sigma}_{0} \\ &\quad=\text{E}(\boldsymbol{C}\boldsymbol{y}-\widetilde{\boldsymbol{C}}\boldsymbol{y})'(\boldsymbol{C}\boldsymbol{y}-\widetilde{\boldsymbol{C}}\boldsymbol{y})+\text{R}( \widetilde{\boldsymbol{C}}\boldsymbol{y}) \\ &\qquad{}+\text{E}\bigl\{ 2{\lambda}(\boldsymbol{C}\boldsymbol{y}-\widetilde{\boldsymbol{C}}\boldsymbol{y})'(\widetilde{\boldsymbol{C}}\boldsymbol{y}-\boldsymbol{y}_{0}) +2(1-{\lambda}) (\boldsymbol{C}\boldsymbol{y}-\widetilde{\boldsymbol{C}}\boldsymbol{y})'(\widetilde{\boldsymbol{C}}\boldsymbol{y}-\boldsymbol{X}_{0}\boldsymbol{\beta}) \bigr\} . \end{aligned}$$

Note that

$$\begin{aligned} &\text{E}\boldsymbol{y}\boldsymbol{y}'=\boldsymbol{\Sigma}+\boldsymbol{X}\boldsymbol{\beta} \boldsymbol{\beta}'\boldsymbol{X}'=\boldsymbol{T}-\boldsymbol{X}\boldsymbol{X}'+\boldsymbol{X}\boldsymbol{\beta} \boldsymbol{\beta}'\boldsymbol{X}', \\ &\text{E}\boldsymbol{y}_{0}\boldsymbol{y}'=\boldsymbol{V}+\boldsymbol{X}_{0}\boldsymbol{\beta} \boldsymbol{\beta}'\boldsymbol{X}', \\ &(\boldsymbol{C}-\widetilde{\boldsymbol{C}})\boldsymbol{X}=\bigl(\boldsymbol{C}-{\lambda} \boldsymbol{V}\boldsymbol{T}^{+}\bigr)\bigl[\boldsymbol{I}-\boldsymbol{X}\bigl(\boldsymbol{X}'\boldsymbol{T}^{+}\boldsymbol{X}\bigr)^{-}\boldsymbol{X}'\boldsymbol{T}^{+}\bigr]\boldsymbol{X}=\mathbf{0}, \end{aligned}$$

then

$$\begin{aligned} &\text{E}\bigl\{ 2{\lambda}(\boldsymbol{C}\boldsymbol{y}-\widetilde{\boldsymbol{C}}\boldsymbol{y})'(\widetilde{\boldsymbol{C}}\boldsymbol{y}-\boldsymbol{y}_{0}) +2(1-{\lambda}) (\boldsymbol{C}\boldsymbol{y}- \widetilde{\boldsymbol{C}}\boldsymbol{y})'(\widetilde{ \boldsymbol{C}}\boldsymbol{y}-\boldsymbol{X}_{0}\boldsymbol{\beta})\bigr\} \\ &\quad=\text{E}\bigl\{ 2(\boldsymbol{C}\boldsymbol{y}-\widetilde{\boldsymbol{C}}\boldsymbol{y})'(\boldsymbol{C}\boldsymbol{y}-\boldsymbol{y}_{0})+2(1-{\lambda}) (\boldsymbol{C}\boldsymbol{y}- \widetilde{\boldsymbol{C}}\boldsymbol{y})'(\boldsymbol{y}_{0}-\boldsymbol{X}_{0}\boldsymbol{\beta})\bigr\} \\ &\quad=\operatorname{tr}\bigl\{ \bigl[\boldsymbol{C}\boldsymbol{X}\bigl(\boldsymbol{X}'\boldsymbol{T}^{+}\boldsymbol{X}\bigr)^{-}\boldsymbol{X}'-{\lambda} \boldsymbol{V}\boldsymbol{T}^{+}\bigl(\boldsymbol{X}'\boldsymbol{T}^{+}\boldsymbol{X}\bigr)^{-}\boldsymbol{X}'-\boldsymbol{C}\boldsymbol{X}\boldsymbol{X}'+\boldsymbol{C}\boldsymbol{X}\boldsymbol{\beta} \boldsymbol{\beta}'\boldsymbol{X}'-\boldsymbol{X}_{0}\boldsymbol{\beta} \boldsymbol{\beta}'\boldsymbol{X}'\bigr] (\boldsymbol{C}-\widetilde{\boldsymbol{C}})'\bigr\} \\ &\quad=\operatorname{tr}\bigl\{ \bigl[\boldsymbol{C}\boldsymbol{X}\bigl(\boldsymbol{X}'\boldsymbol{T}^{+}\boldsymbol{X}\bigr)^{-}-{\lambda} \boldsymbol{V}\boldsymbol{T}^{+}\bigl(\boldsymbol{X}'\boldsymbol{T}^{+}\boldsymbol{X}\bigr)^{-}-\boldsymbol{C}\boldsymbol{X}+\boldsymbol{C}\boldsymbol{X}\boldsymbol{\beta} \boldsymbol{\beta}'- \boldsymbol{X}_{0}\boldsymbol{\beta} \boldsymbol{\beta}'\bigr] \bigl[(\boldsymbol{C}-\widetilde{\boldsymbol{C}})\boldsymbol{X}\bigr]'\bigr\} \\ &\quad=0. \end{aligned}$$

Thus, we have

$$\begin{aligned} \text{R}(\boldsymbol{C}\boldsymbol{y};\boldsymbol{\beta})& =\text{E}(\boldsymbol{C}\boldsymbol{y}-\widetilde{\boldsymbol{C}}\boldsymbol{y})'(C\boldsymbol{y}- \widetilde{\boldsymbol{C}}\boldsymbol{y})+\text{R}(\widetilde{\boldsymbol{C}}\boldsymbol{y};\boldsymbol{\beta}) \geq \text{R}(\widetilde{\boldsymbol{C}}\boldsymbol{y};\boldsymbol{\beta}), \end{aligned}$$

and the equation holds for every $\boldsymbol{\beta}\in\mathbb{R}^{p}$ if and only if

$$\begin{aligned}\text{E}(\boldsymbol{C}\boldsymbol{y}-\widetilde{\boldsymbol{C}}\boldsymbol{y})'(C\boldsymbol{y}-\widetilde{ \boldsymbol{C}}\boldsymbol{y})=0.\end{aligned}$$

Since

$$\begin{aligned} &\text{E}(\boldsymbol{C}\boldsymbol{y}-\widetilde{\boldsymbol{C}}\boldsymbol{y})'(\boldsymbol{C}\boldsymbol{y}-\widetilde{\boldsymbol{C}}\boldsymbol{y})=0 \\ &\quad\Leftrightarrow\quad\bigl[(\boldsymbol{C}-\widetilde{\boldsymbol{C}})\boldsymbol{X}\boldsymbol{\beta}\bigr]' \bigl[(\boldsymbol{C}- \widetilde{\boldsymbol{C}})X\boldsymbol{\beta}\bigr] +\operatorname{tr}(\boldsymbol{C}-\widetilde{\boldsymbol{C}})'\boldsymbol{\Sigma}(\boldsymbol{C}- \widetilde{\boldsymbol{C}})=0 \\ &\quad\Leftrightarrow\quad(\boldsymbol{C}-\widetilde{\boldsymbol{C}})\boldsymbol{\Sigma}^{\frac {1}{2}}= \mathbf{0} \\ &\quad\Leftrightarrow\quad(\boldsymbol{C}-\widetilde{\boldsymbol{C}})\boldsymbol{\Sigma}=\mathbf{0}, \end{aligned}$$

the equation holds for every $\boldsymbol{\beta}\in\mathbb{R}^{p}$ if and only if

$$C\boldsymbol{\Sigma}=\widetilde{\boldsymbol{C}}\boldsymbol{\Sigma}. $$

Now we prove conditions (1) and (2) are equivalent. First, as $\boldsymbol{C}\boldsymbol{\Sigma}=\widetilde{\boldsymbol{C}}\boldsymbol{\Sigma}$,

$$\begin{aligned} &\boldsymbol{\Sigma} \boldsymbol{C}' =\boldsymbol{\Sigma}\widetilde{\boldsymbol{C}}' \\ &\hphantom{\boldsymbol{\Sigma} \boldsymbol{C}'} =\boldsymbol{\Sigma} \boldsymbol{T}^{+}\boldsymbol{X}\bigl(\boldsymbol{X}'\boldsymbol{T}^{+}\boldsymbol{X}\bigr)^{-}\boldsymbol{X}'\boldsymbol{C}'+{\lambda} \boldsymbol{\Sigma}\bigl[\boldsymbol{I}-\boldsymbol{X}\bigl(\boldsymbol{X}'\boldsymbol{T}^{+}\boldsymbol{X}\bigr)^{-}\boldsymbol{X}'\boldsymbol{T}^{+}\bigr]'\boldsymbol{T}^{+}\boldsymbol{V}' \\ & \hphantom{\boldsymbol{\Sigma} \boldsymbol{C}'}=\bigl(\boldsymbol{T}-\boldsymbol{X}\boldsymbol{X}'\bigr)\boldsymbol{T}^{+}\boldsymbol{X}\bigl(\boldsymbol{X}'\boldsymbol{T}^{+}\boldsymbol{X}\bigr)^{-}\boldsymbol{X}'\boldsymbol{C}' \\ &\hphantom{\boldsymbol{\Sigma} \boldsymbol{C}'=}+{\lambda}\bigl(\boldsymbol{T}-\boldsymbol{X}\boldsymbol{X}'\bigr)\bigl[\boldsymbol{I}-\boldsymbol{X}\bigl(\boldsymbol{X}'\boldsymbol{T}^{+}\boldsymbol{X}\bigr)^{-}\boldsymbol{X}'\boldsymbol{T}^{+} \bigr]'\boldsymbol{T}^{+}\boldsymbol{V}' \\ &\hphantom{\boldsymbol{\Sigma} \boldsymbol{C}'} =\bigl[\boldsymbol{X}\bigl(\boldsymbol{X}'\boldsymbol{T}^{+}\boldsymbol{X}\bigr)^{-}\boldsymbol{X}'-\boldsymbol{X}\boldsymbol{X}'\bigr]+{\lambda} \boldsymbol{V}'-{\lambda} \boldsymbol{X}\bigl(\boldsymbol{X}'\boldsymbol{T}^{+}\boldsymbol{X}\bigr)^{-}\boldsymbol{X}'\boldsymbol{T}^{+}\boldsymbol{V}'\quad\Rightarrow \\ &\boldsymbol{\Sigma} \boldsymbol{C}'-{\lambda} \boldsymbol{V}' =\boldsymbol{X}\bigl[\bigl(\boldsymbol{X}'\boldsymbol{T}^{+}\boldsymbol{X}\bigr)^{-}\boldsymbol{X}'\boldsymbol{C}'-\boldsymbol{X}'\boldsymbol{C}'-{\lambda}\bigl(\boldsymbol{X}'\boldsymbol{T}^{+}\boldsymbol{X}\bigr)^{-}\boldsymbol{X}'\boldsymbol{T}^{+}\boldsymbol{V}'\bigr], \end{aligned}$$

which shows that $\mathscr{M}(\boldsymbol{\Sigma} \boldsymbol{C}'-{\lambda} \boldsymbol{V}')\subseteq \mathscr{M}(\boldsymbol{X})$.

Second, as $\mathscr{M}(\Sigma \boldsymbol{C}'-{\lambda} \boldsymbol{V}')\subseteq\mathscr{M}(\boldsymbol{X})$, there exists a matrix ***H*** such that

$$\boldsymbol{\Sigma} \boldsymbol{C}'-{\lambda} \boldsymbol{V}'=\boldsymbol{X}\boldsymbol{H}, \quad\mbox{or} \quad {\lambda} \boldsymbol{V}=\boldsymbol{C}\boldsymbol{\Sigma}+\boldsymbol{H}'\boldsymbol{X}'. $$

Then

$$\begin{aligned} \widetilde{\boldsymbol{C}}\Sigma={}&\boldsymbol{C}\boldsymbol{X}\bigl(\boldsymbol{X}'\boldsymbol{T}^{+}\boldsymbol{X}\bigr)^{-}\boldsymbol{X}'\boldsymbol{T}^{+}\bigl(\boldsymbol{T}-\boldsymbol{X}\boldsymbol{X}'\bigr)+ \lambda \boldsymbol{V}\boldsymbol{T}^{+}\bigl[\boldsymbol{I}-\boldsymbol{X}\bigl(\boldsymbol{X}'\boldsymbol{T}^{+}\boldsymbol{X}\bigr)^{-}\boldsymbol{X}'\boldsymbol{T}^{+}\bigr]\bigl(\boldsymbol{T}-\boldsymbol{X}\boldsymbol{X}'\bigr) \\ ={}&\bigl(\boldsymbol{C}\boldsymbol{\Sigma}-\boldsymbol{H}'\boldsymbol{X}'\bigr)\boldsymbol{T}^{+} \bigl[\boldsymbol{I}-\boldsymbol{X}\bigl(\boldsymbol{X}'\boldsymbol{T}^{+}\boldsymbol{X}\bigr)^{-}\boldsymbol{X}'\boldsymbol{T}^{+}\bigr]\bigl(\boldsymbol{T}-\boldsymbol{X}\boldsymbol{X}'\bigr) \\ &{}+\boldsymbol{C}\boldsymbol{X}\bigl(\boldsymbol{X}'\boldsymbol{T}^{+}\boldsymbol{X}\bigr)^{-}\boldsymbol{X}'-\boldsymbol{C}\boldsymbol{X}\boldsymbol{X}' \\ ={}&\boldsymbol{C}\boldsymbol{X}\bigl(\boldsymbol{X}'\boldsymbol{T}^{+}\boldsymbol{X}\bigr)^{-}\boldsymbol{X}'-\boldsymbol{C}\boldsymbol{X}\boldsymbol{X}'+C\boldsymbol{\Sigma}-C\bigl(\boldsymbol{T}-\boldsymbol{X}\boldsymbol{X}' \bigr)\boldsymbol{T}^{+}\boldsymbol{X}\bigl(\boldsymbol{X}'\boldsymbol{T}^{+}\boldsymbol{X}\bigr)^{-}\boldsymbol{X}' \\ ={}&\boldsymbol{C}\boldsymbol{\Sigma}. \end{aligned}$$

Therefore, we complete the proof. □

### Lemma 2.4

*If* $\boldsymbol{C}\boldsymbol{\Sigma}=\widetilde{\boldsymbol{C}}\boldsymbol{\Sigma}$, *then the risk of* ***Cy*** *under the quadratic loss function is*

$$\begin{aligned} \text{R}(\boldsymbol{C}\boldsymbol{y};\boldsymbol{\beta}) ={}&\text{R}(\widetilde{\boldsymbol{C}}\boldsymbol{y};\boldsymbol{\beta})=\operatorname{tr}\bigl[\boldsymbol{C}\boldsymbol{X}\boldsymbol{Q}\boldsymbol{X}'\boldsymbol{C}'+{\lambda}^{2} \boldsymbol{\Sigma}_{0}-2{\lambda} \boldsymbol{C}\boldsymbol{X}\bigl(\boldsymbol{X}'\boldsymbol{T}^{+}\boldsymbol{X}\bigr)^{-}\boldsymbol{X}'\boldsymbol{T}^{+}\boldsymbol{V}' \\ &{} -{\lambda}^{2}\boldsymbol{V}\bigl(\boldsymbol{T}^{+}-\boldsymbol{T}^{+}\boldsymbol{X}\bigl(\boldsymbol{X}'\boldsymbol{T}^{+}\boldsymbol{X}\bigr)^{-}\boldsymbol{X}'\boldsymbol{T}^{+}\bigr)\boldsymbol{V}'\bigr]+\boldsymbol{\beta}'(\boldsymbol{C}\boldsymbol{X}-\boldsymbol{X}_{0})'(\boldsymbol{C}\boldsymbol{X}-\boldsymbol{X}_{0})\boldsymbol{\beta}, \end{aligned}$$

*where* $\boldsymbol{Q}=(\boldsymbol{X}'\boldsymbol{T}^{+}\boldsymbol{X})^{-}-\boldsymbol{I}=(\boldsymbol{X}'\boldsymbol{T}^{+}\boldsymbol{X})^{-}\boldsymbol{X}'\boldsymbol{T}^{+}\boldsymbol{\Sigma} \boldsymbol{T}^{+}\boldsymbol{X}(\boldsymbol{X}'\boldsymbol{T}^{+}\boldsymbol{X})^{-}$.

### Proof

From Lemma 2.3, when $C\Sigma=\widetilde{C} \Sigma$,

$$\begin{aligned} \text{R}(\boldsymbol{C}\boldsymbol{y};\boldsymbol{\beta}) &=\text{R}(\widetilde{\boldsymbol{C}}\boldsymbol{y};\boldsymbol{\beta})=\operatorname{tr}\bigl( \widetilde{\boldsymbol{C}} \boldsymbol{\Sigma}\widetilde{\boldsymbol{C}}'+{\lambda}^{2} \boldsymbol{\Sigma}_{0}-2 {\lambda}\widetilde{\boldsymbol{C}}\boldsymbol{V}'\bigr)+\boldsymbol{\beta}'(\widetilde{\boldsymbol{C}}\boldsymbol{X}-\boldsymbol{X}_{0})'( \widetilde{\boldsymbol{C}}\boldsymbol{X}-\boldsymbol{X}_{0})\boldsymbol{\beta}. \end{aligned}$$

Since $\widetilde{\boldsymbol{C}}=\boldsymbol{C}\boldsymbol{X}(\boldsymbol{X}'\boldsymbol{T}^{+}\boldsymbol{X})^{-}\boldsymbol{X}'\boldsymbol{T}^{+}+{\lambda} \boldsymbol{V}\boldsymbol{T}^{+}[\boldsymbol{I}-\boldsymbol{X}(\boldsymbol{X}'\boldsymbol{T}^{+}\boldsymbol{X})^{-}\boldsymbol{X}'\boldsymbol{T}^{+}]$ and $\boldsymbol{\Sigma}=\boldsymbol{T}-\boldsymbol{X}\boldsymbol{X}'$,

$$\begin{aligned} &\widetilde{\boldsymbol{C}}\Sigma\widetilde{\boldsymbol{C}}' =\boldsymbol{C}\boldsymbol{X}\boldsymbol{Q}\boldsymbol{X}' \boldsymbol{C}'+{\lambda}^{2}\boldsymbol{V}\bigl(\boldsymbol{T}^{+}-\boldsymbol{T}^{+}\boldsymbol{X}\bigl(\boldsymbol{X}'\boldsymbol{T}^{+}\boldsymbol{X}\bigr)^{-}\boldsymbol{X}' \boldsymbol{T}^{+}\bigr)\boldsymbol{V}', \\ &\boldsymbol{Q}=\bigl(\boldsymbol{X}'\boldsymbol{T}^{+}\boldsymbol{X}\bigr)^{-}-\boldsymbol{I}=\bigl( \boldsymbol{X}'\boldsymbol{T}^{+}\boldsymbol{X}\bigr)^{-}\boldsymbol{X}'\boldsymbol{T}^{+}\boldsymbol{\Sigma} \boldsymbol{T}^{+}\boldsymbol{X}\bigl(\boldsymbol{X}'\boldsymbol{T}^{+}\boldsymbol{X}\bigr)^{-}, \\ &\widetilde{\boldsymbol{C}}\boldsymbol{V}' =\boldsymbol{C}\boldsymbol{X}\bigl(\boldsymbol{X}'\boldsymbol{T}^{+}\boldsymbol{X}\bigr)^{-}\boldsymbol{X}'\boldsymbol{T}^{+}\boldsymbol{V}'+{\lambda} \boldsymbol{V}\boldsymbol{T}^{+}\bigl[\boldsymbol{I}-\boldsymbol{X}\bigl(\boldsymbol{X}'\boldsymbol{T}^{+}\boldsymbol{X}\bigr)^{-}\boldsymbol{X}'\boldsymbol{T}^{+}\bigr]\boldsymbol{V}', \\ &\widetilde{\boldsymbol{C}}\boldsymbol{X}=\boldsymbol{C}\boldsymbol{X}. \end{aligned}$$

The lemma is easily proved by substitution of these equations. □

## Admissibility of homogeneous linear predictors

In this section, we derive the necessary and sufficient conditions for the admissibility of simultaneous prediction in class of the homogeneous linear predictors. The best linear unbiased predictor of ***δ*** is obtained. Examples are presented to give some admissible predictors.

### Theorem 3.1

*Let* $\boldsymbol{l}'=\boldsymbol{q}'\boldsymbol{C}$, *where* ***C*** *is a matrix and* ***l***, ***q*** *are vectors with appropriate dimensions*. *If* $\boldsymbol{C}\boldsymbol{y}\sim \boldsymbol{\delta}$ *under the quadratic loss function*, *then*

$$\boldsymbol{l}'\boldsymbol{X}\boldsymbol{Q}\boldsymbol{X}'\boldsymbol{l}\leq {\lambda}\bigl(\boldsymbol {l}'\boldsymbol{X}- \boldsymbol{q}'\boldsymbol{X}_{0}\bigr) \bigl(\boldsymbol{X}'\boldsymbol{T}^{+} \boldsymbol{X}\bigr)^{-}\boldsymbol{X}'\boldsymbol{T}^{+}\boldsymbol{V}'\boldsymbol{q}+\boldsymbol{l} '\boldsymbol{X}\boldsymbol{Q}\boldsymbol{X}'_{0}\boldsymbol{q}, $$

*where* $\boldsymbol{Q}=(\boldsymbol{X}'\boldsymbol{T}^{+}\boldsymbol{X})^{-}-\boldsymbol{I}=(\boldsymbol{X}'\boldsymbol{T}^{+}\boldsymbol{X})^{-}\boldsymbol{X}'\boldsymbol{T}^{+} \Sigma \boldsymbol{T}^{+}\boldsymbol{X}(\boldsymbol{X}'\boldsymbol{T}^{+}\boldsymbol{X})^{-}$.

### Proof

Since $\mathbf{C\boldsymbol{y}} \sim \boldsymbol{\delta}$ and $\boldsymbol{l}'=\boldsymbol{q}'\boldsymbol{C}$, by Lemma 2.1, $\boldsymbol {l}'\boldsymbol{y}\sim\boldsymbol{q}'\boldsymbol{\delta}$ for any $1\times m$ vector ***q***. Suppose *k* be a real constant and $0< k<1$. Let

$$\boldsymbol{l}_{k}'=\bigl[k\boldsymbol{l}'\boldsymbol{X}+(1-k)\boldsymbol{q} '\boldsymbol{X}_{0}\bigr]\bigl(\boldsymbol{X}'\boldsymbol{T}^{+}\boldsymbol{X}\bigr)^{-}\boldsymbol{X}'\boldsymbol{T}^{+}+{\lambda}\boldsymbol{q}'\boldsymbol{V}\boldsymbol{T}^{+}\bigl[I-\boldsymbol{X}\bigl(\boldsymbol{X}'\boldsymbol{T}^{+}\boldsymbol{X}\bigr)^{-}\boldsymbol{X}'\boldsymbol{T}^{+}\bigr]. $$

The risk of $\boldsymbol{l}_{k}'\boldsymbol{y}$ is

$$\begin{aligned} \text{R}\bigl(\boldsymbol{l}_{k}'\boldsymbol{y};\boldsymbol{\beta}\bigr) ={}& \bigl[k\boldsymbol{l}'\boldsymbol{X}+(1-k)\boldsymbol{q}'\boldsymbol{X}_{0}\bigr]\boldsymbol{Q}\bigl[k\boldsymbol{l}'\boldsymbol{X}+(1-k)\boldsymbol{q}'\boldsymbol{X}_{0} \bigr]'+{\lambda}^{2} \boldsymbol{q}'\boldsymbol{\Sigma}_{0}\boldsymbol{q} \\ &{}-2{\lambda}\bigl[k\boldsymbol{l}'\boldsymbol{X}+(1-k)\boldsymbol{q}'\boldsymbol{X}_{0} \bigr]\bigl(\boldsymbol{X}'\boldsymbol{T}^{+}\boldsymbol{X}\bigr)^{-}\boldsymbol{X}'\boldsymbol{T}^{+}\boldsymbol{V}'\boldsymbol{q} \\ &{}-{\lambda}^{2}\boldsymbol{q}'\boldsymbol{V}\boldsymbol{T}^{+}\bigl[\boldsymbol{I}-\boldsymbol{X}\bigl(\boldsymbol{X}'\boldsymbol{T}^{+}\boldsymbol{X}\bigr)^{-}\boldsymbol{X}'\boldsymbol{T}^{+}\bigr]\boldsymbol{V}'\boldsymbol{q}+k^{2}\bigl[\boldsymbol{\beta}'\bigl(\boldsymbol{l}'\boldsymbol{X}-\boldsymbol{q}'\boldsymbol{X}_{0} \bigr)'\bigr]^{2}. \end{aligned}$$

Since $\boldsymbol{l}'\boldsymbol{y}\sim\boldsymbol{q}'\boldsymbol{y}_{0}$ and by Lemma 2.3, Lemma 2.4 and $\boldsymbol{l}'=\boldsymbol{q}'\boldsymbol{C}$, the risk of $\boldsymbol {l}'\boldsymbol{y}$ is

$$\begin{aligned} \text{R}\bigl(\boldsymbol{l}'\boldsymbol{y};\boldsymbol{\beta}\bigr) ={}&\boldsymbol{l} '\boldsymbol{X}\boldsymbol{Q}\boldsymbol{X}'l+{\lambda}^{2} \boldsymbol{q}' \boldsymbol{\Sigma}_{0}\boldsymbol{q}-2{\lambda}\boldsymbol{l}'\boldsymbol{X}\bigl(\boldsymbol{X}'\boldsymbol{T}^{+}\boldsymbol{X}\bigr)^{-}\boldsymbol{X}'\boldsymbol{T}^{+}\boldsymbol{V}'\boldsymbol{q} \\ &{} -{\lambda}^{2}\boldsymbol{q}'\boldsymbol{V}\bigl(\boldsymbol{T}^{+}-\boldsymbol{T}^{+}\boldsymbol{X}\bigl(\boldsymbol{X}'\boldsymbol{T}^{+}\boldsymbol{X}\bigr)^{-} \boldsymbol{X}'\boldsymbol{T}^{+}\bigr)\boldsymbol{V}'\boldsymbol{q}+\bigl[\boldsymbol{\beta}'\bigl(\boldsymbol{l}'\boldsymbol{X}-\boldsymbol{q}'\boldsymbol{X}_{0} \bigr)'\bigr]^{2}, \end{aligned}$$

and for any $k\in(0,1)$, $\text{R}(\boldsymbol{l}'\boldsymbol{y}; \boldsymbol{\beta})\leq \text{R}(\boldsymbol {l}_{k}'\boldsymbol{y}; \boldsymbol{\beta})$. It means that

$$\begin{aligned} &\text{R}\bigl(\boldsymbol{l}'\boldsymbol{y};\boldsymbol{\beta}\bigr)-\text{R}\bigl(\boldsymbol{l}_{k}' \boldsymbol{y};\boldsymbol{\beta}\bigr) \\ & \quad =\bigl(1-k^{2}\bigr)l'\boldsymbol{X}\boldsymbol{Q}\boldsymbol{X}'l-2(1-k){\lambda}\boldsymbol{l}'\boldsymbol{X}\bigl(\boldsymbol{X}'\boldsymbol{T}^{+}\boldsymbol{X}\bigr)^{-}\boldsymbol{X}'\boldsymbol{T}^{+}\boldsymbol{V}'\boldsymbol{q} \\ &\qquad {}-2k(1-k)\boldsymbol{q}'\boldsymbol{X}_{0}\boldsymbol{Q}\boldsymbol{X}'l-(1-k)^{2} \boldsymbol{q}'\boldsymbol{X}_{0}\boldsymbol{Q}\boldsymbol{X}_{0}'\boldsymbol{q} \\ &\qquad {}+2(1-k){\lambda}\boldsymbol{q}'\boldsymbol{X}_{0}\bigl(\boldsymbol{X}'\boldsymbol{T}^{+}\boldsymbol{X}\bigr)^{-}\boldsymbol{X}'\boldsymbol{T}^{+}\boldsymbol{V}'\boldsymbol{q}\leq0. \end{aligned}$$

Divide both sides of the above inequality by $1-k$ and then let $k\rightarrow1$, we have

$$\boldsymbol{l}'\boldsymbol{X}\boldsymbol{Q}\boldsymbol{X}'l\leq {\lambda}\bigl(\boldsymbol{l}'\boldsymbol{X}-\boldsymbol{q} '\boldsymbol{X}_{0}\bigr) \bigl(\boldsymbol{X}'\boldsymbol{T}^{+}\boldsymbol{X}\bigr)^{-}\boldsymbol{X}'\boldsymbol{T}^{+}\boldsymbol{V}'\boldsymbol{q}+\boldsymbol{l} '\boldsymbol{X}\boldsymbol{Q}\boldsymbol{X}'_{0}\boldsymbol{q}. $$

 □

### Theorem 3.2

*For the model* (3), $\boldsymbol{C}\boldsymbol{y}\sim \boldsymbol{\delta}$ *in* $\mathscr{LH}$ *under the quadratic loss function if and only if*

1. $\boldsymbol{C}\boldsymbol{\Sigma}=\widetilde{\boldsymbol{C}}\boldsymbol{\Sigma}$ (*equivalently* $\mathscr{M}(\boldsymbol{\Sigma} \boldsymbol{C}'-{\lambda} \boldsymbol{V}')\subseteq\mathscr{M}(\boldsymbol{X})$), *and*
2. $\boldsymbol{C}\boldsymbol{X}\boldsymbol{Q}\boldsymbol{X}'\boldsymbol{C}'\leq {\lambda}(\boldsymbol{C}\boldsymbol{X}-\boldsymbol{X}_{0})(\boldsymbol{X}'\boldsymbol{T}^{+}\boldsymbol{X})^{-}\boldsymbol{X}'\boldsymbol{T}^{+}\boldsymbol{V}'+\boldsymbol{C}\boldsymbol{X}\boldsymbol{Q}\boldsymbol{X}'_{0}$, *where* $\boldsymbol{Q}=(\boldsymbol{X}'\boldsymbol{T}^{+}\boldsymbol{X})^{-}-\boldsymbol{I}=(\boldsymbol{X}'\boldsymbol{T}^{+}\boldsymbol{X})^{-}\boldsymbol{X}'\boldsymbol{T}^{+}\boldsymbol{\Sigma} \boldsymbol{T}^{+}\boldsymbol{X}(\boldsymbol{X}'\boldsymbol{T}^{+}\boldsymbol{X})^{-}$, *and*
3. $\mathscr{M}(\boldsymbol{C}\boldsymbol{X}-\boldsymbol{X}_{0})=\mathscr{M}(\boldsymbol{G})$, *where* $\boldsymbol{G}=(\boldsymbol{C}\boldsymbol{X}-\boldsymbol{X}_{0})\boldsymbol{Q}(\boldsymbol{C}\boldsymbol{X}-\boldsymbol{X}_{0})'+(\boldsymbol{C}\boldsymbol{X}-\boldsymbol{X}_{0})[{\lambda}(\boldsymbol{X}'\boldsymbol{T}^{+}\boldsymbol{X})^{-}\boldsymbol{X}'\boldsymbol{T}^{+}\boldsymbol{V}'-\boldsymbol{Q}\boldsymbol{X}'\boldsymbol{C}']$.

### Proof

Necessity:

(i) The condition (1) is shown in Lemma 2.3;
(ii) Since $\boldsymbol{C}\boldsymbol{y}\sim \boldsymbol{\delta}$, then $\boldsymbol{\eta}'\boldsymbol{C}\boldsymbol{y}\sim \boldsymbol{\eta}'\boldsymbol{\delta}$ for any $\boldsymbol{\eta}\in\mathbb{R}^{m}$ by Lemma 2.2. Let $\boldsymbol{l}'=\boldsymbol{\eta}'\boldsymbol{C}$ and $\boldsymbol{q}'=\boldsymbol{\eta}'$ in Theorem 3.1, we have
  4 $$ \boldsymbol{\eta}'\boldsymbol{C}\boldsymbol{X}\boldsymbol{Q}\boldsymbol{X}'\boldsymbol{C}' \boldsymbol{\eta}\leq {\lambda} \boldsymbol{\eta}'(\boldsymbol{C}\boldsymbol{X}-\boldsymbol{X}_{0}) \bigl(\boldsymbol{X}'\boldsymbol{T}^{+}\boldsymbol{X}\bigr)^{-}\boldsymbol{X}'\boldsymbol{T}^{+}\boldsymbol{V}'\boldsymbol{\eta}+\boldsymbol{\eta}'\boldsymbol{C}\boldsymbol{X}\boldsymbol{Q}\boldsymbol{X}'_{0}\boldsymbol{\eta}. $$

To prove condition (2), we can only prove that ${\lambda}(\boldsymbol{C}\boldsymbol{X}-\boldsymbol{X}_{0})(\boldsymbol{X}'\boldsymbol{T}^{+}\boldsymbol{X})^{-}\boldsymbol{X}'\boldsymbol{T}^{+}\boldsymbol{V}'+\boldsymbol{C}\boldsymbol{X}\boldsymbol{Q}\boldsymbol{X}'_{0}$ is symmetric from (4). Using reduction to absurdity and suppose ${\lambda}(\boldsymbol{C}\boldsymbol{X}-\boldsymbol{X}_{0})(\boldsymbol{X}'\boldsymbol{T}^{+}\boldsymbol{X})^{-}\boldsymbol{X}'\boldsymbol{T}^{+}\boldsymbol{V}'+\boldsymbol{C}\boldsymbol{X}\boldsymbol{Q}\boldsymbol{X}'_{0}$ is not a symmetric matrix. With this assumption and the fact that $\boldsymbol{C}\boldsymbol{X}\boldsymbol{Q}\boldsymbol{X}'\boldsymbol{C}'$ and $(\boldsymbol{C}\boldsymbol{X}-\boldsymbol{X}_{0})\boldsymbol{Q}(\boldsymbol{C}\boldsymbol{X}-\boldsymbol{X}_{0})'$ are both symmetric positive semi-definite matrices, we have

$$\begin{aligned} &{\lambda}(\boldsymbol{C}\boldsymbol{X}-\boldsymbol{X}_{0}) \bigl(\boldsymbol{X}'\boldsymbol{T}^{+}\boldsymbol{X}\bigr)^{-}\boldsymbol{X}'\boldsymbol{T}^{+}\boldsymbol{V}'+\boldsymbol{C}\boldsymbol{X}\boldsymbol{Q}\boldsymbol{X}'_{0}-\boldsymbol{C}\boldsymbol{X}\boldsymbol{Q}\boldsymbol{X}'\boldsymbol{C}' \\ &\quad=-(\boldsymbol{C}\boldsymbol{X}-\boldsymbol{X}_{0})\boldsymbol{Q}\boldsymbol{X}_{0}'-(\boldsymbol{C}\boldsymbol{X}-\boldsymbol{X}_{0})\boldsymbol{Q}(\boldsymbol{C}\boldsymbol{X}-\boldsymbol{X}_{0})' \\ &\qquad {}+{\lambda}(\boldsymbol{C}\boldsymbol{X}-\boldsymbol{X}_{0}) \bigl(\boldsymbol{X}'\boldsymbol{T}^{+}\boldsymbol{X}\bigr)^{-} \boldsymbol{X}'\boldsymbol{T}^{+}\boldsymbol{V}' \end{aligned}$$

is not symmetric and hence

$$-(\boldsymbol{C}\boldsymbol{X}-\boldsymbol{X}_{0})\boldsymbol{Q}\boldsymbol{X}_{0}'+{\lambda}(\boldsymbol{C}\boldsymbol{X}-\boldsymbol{X}_{0}) \bigl(\boldsymbol{X}'\boldsymbol{T}^{+}\boldsymbol{X}\bigr)^{-} \boldsymbol{X}'\boldsymbol{T}^{+}\boldsymbol{V}' $$

is not symmetric. By Lemma 2.2, there exists an orthogonal matrix ***P*** such that

5 $$\begin{aligned} &\operatorname{tr}\bigl\{ \boldsymbol{P}\bigl[-(\boldsymbol{C}\boldsymbol{X}-\boldsymbol{X}_{0})\boldsymbol{Q}\boldsymbol{X}_{0}'+{\lambda}(\boldsymbol{C}\boldsymbol{X}-\boldsymbol{X}_{0}) \bigl(\boldsymbol{X}'\boldsymbol{T}^{+}\boldsymbol{X}\bigr)^{-}\boldsymbol{X}'\boldsymbol{T}^{+}\boldsymbol{V}'\bigr]\bigr\} \\ &\quad>\operatorname{tr}\bigl[-(\boldsymbol{C}\boldsymbol{X}-\boldsymbol{X}_{0})\boldsymbol{Q}\boldsymbol{X}_{0}'+{\lambda}(\boldsymbol{C}\boldsymbol{X}-\boldsymbol{X}_{0}) \bigl(\boldsymbol{X}'\boldsymbol{T}^{+}\boldsymbol{X}\bigr)^{-}\boldsymbol{X}'\boldsymbol{T}^{+}\boldsymbol{V}'\bigr]. \end{aligned}$$

Let

$$\begin{aligned} \boldsymbol{H} =&\boldsymbol{X}_{0}\bigl(\boldsymbol{X}'\boldsymbol{T}^{+}\boldsymbol{X}\bigr)^{-}\boldsymbol{X}'\boldsymbol{T}^{+}+{\lambda} \boldsymbol{V}\boldsymbol{T}^{+}\bigl[\boldsymbol{I}-\boldsymbol{X}\bigl(\boldsymbol{X}'\boldsymbol{T}^{+}\boldsymbol{X}\bigr)^{-}\boldsymbol{X}'\boldsymbol{T}^{+}\bigr] \\ &{}-P\bigl\{ \boldsymbol{X}_{0}\bigl(\boldsymbol{X}'\boldsymbol{T}^{+}\boldsymbol{X}\bigr)^{-}\boldsymbol{X}'\boldsymbol{T}^{+}+{\lambda} \boldsymbol{V}\boldsymbol{T}^{+} \bigl[\boldsymbol{I}-\boldsymbol{X}\bigl(\boldsymbol{X}'\boldsymbol{T}^{+}\boldsymbol{X}\bigr)^{-}\boldsymbol{X}'\boldsymbol{T}^{+}\bigr]-\boldsymbol{C}\bigr\} \end{aligned}$$

and

$$\begin{aligned}\widetilde{\boldsymbol{H}}=\boldsymbol{H}\boldsymbol{X}\bigl(\boldsymbol{X}'\boldsymbol{T}^{+}\boldsymbol{X}\bigr)^{-}\boldsymbol{X}'\boldsymbol{T}^{+}+{\lambda} \boldsymbol{V}\boldsymbol{T}^{+}\bigl[\boldsymbol{I}-\boldsymbol{X}\bigl(\boldsymbol{X}'\boldsymbol{T}^{+}\boldsymbol{X}\bigr)^{-}\boldsymbol{X}'\boldsymbol{T}^{+}\bigr].\end{aligned}$$

It is easy to prove that $\boldsymbol{H}\boldsymbol{\Sigma}=\widetilde {\boldsymbol{H}}\boldsymbol{\Sigma}$. Then with Lemma 2.4 and (5), we have

$$\begin{aligned} \text{R}(\boldsymbol{H}\boldsymbol{y};\boldsymbol{\beta}) =&\text{R}(\widetilde{\boldsymbol{H}}\boldsymbol{y};\boldsymbol{\beta}) \\ =&\operatorname{tr}\bigl\{ (CX-X_{0})Q( \boldsymbol{C}\boldsymbol{X}-\boldsymbol{X}_{0})'+2\boldsymbol{P}(\boldsymbol{C}\boldsymbol{X}-\boldsymbol{X}_{0})\boldsymbol{Q}\boldsymbol{X}_{0}'+\boldsymbol{X}_{0}\boldsymbol{Q}\boldsymbol{X}_{0}' \\ &{}-2{\lambda} \boldsymbol{P}(\boldsymbol{C}\boldsymbol{X}-\boldsymbol{X}_{0}) \bigl(\boldsymbol{X}'\boldsymbol{T}^{+}\boldsymbol{X}\bigr)^{-}\boldsymbol{X}'\boldsymbol{T}^{+}\boldsymbol{V}-2{\lambda} \boldsymbol{X}_{0} \bigl(\boldsymbol{X}'\boldsymbol{T}^{+}\boldsymbol{X}\bigr)^{-}\boldsymbol{X}'\boldsymbol{T}^{+}\boldsymbol{V}+{\lambda}^{2}\boldsymbol{\Sigma}_{0} \\ &{}-{\lambda}^{2}\boldsymbol{V}\boldsymbol{T}^{+}\bigl[\boldsymbol{I}-\boldsymbol{X}\bigl(\boldsymbol{X}'\boldsymbol{T}^{+}\boldsymbol{X}\bigr)^{-}\boldsymbol{X}'\boldsymbol{T}^{+}\bigr] \bigr\} +\boldsymbol{\beta}'(\boldsymbol{C}\boldsymbol{X}-\boldsymbol{X}_{0})'(\boldsymbol{C}\boldsymbol{X}-\boldsymbol{X}_{0})\boldsymbol{\beta}\\ < &\boldsymbol{\beta}'(\boldsymbol{C}\boldsymbol{X}-\boldsymbol{X}_{0})'(\boldsymbol{C}\boldsymbol{X}-\boldsymbol{X}_{0})\boldsymbol{\beta}+\operatorname{tr}\bigl\{ \boldsymbol{C}\boldsymbol{X}\boldsymbol{Q}\boldsymbol{X}'\boldsymbol{C}'-2 {\lambda} \boldsymbol{C}\boldsymbol{X}\bigl(\boldsymbol{X}'\boldsymbol{T}^{+}\boldsymbol{X}\bigr)^{-}\boldsymbol{X}'\boldsymbol{T}^{+}\boldsymbol{V}\\ &{}-{\lambda}^{2}\boldsymbol{V}\boldsymbol{T}^{+}\bigl[\boldsymbol{I}-\boldsymbol{X}\bigl(\boldsymbol{X}'\boldsymbol{T}^{+}\boldsymbol{X}\bigr)^{-}\boldsymbol{X}'\boldsymbol{T}^{+}\bigr]+{\lambda}^{2} \boldsymbol{\Sigma}_{0}\bigr\} \\ =&\text{R}(\boldsymbol{C}\boldsymbol{y};\boldsymbol{\beta}), \end{aligned}$$

which contradicts the admissibility of ***Cy***. Thus,

$$-(\boldsymbol{C}\boldsymbol{X}-\boldsymbol{X}_{0})\boldsymbol{Q}\boldsymbol{X}_{0}'+{\lambda}(\boldsymbol{C}\boldsymbol{X}-\boldsymbol{X}_{0}) \bigl(\boldsymbol{X}'\boldsymbol{T}^{+}\boldsymbol{X}\bigr)^{-} \boldsymbol{X}'\boldsymbol{T}^{+}\boldsymbol{V}' $$

is symmetric and then

$${\lambda}(\boldsymbol{C}\boldsymbol{X}-\boldsymbol{X}_{0}) \bigl(\boldsymbol{X}'\boldsymbol{T}^{+}\boldsymbol{X}\bigr)^{-}\boldsymbol{X}'\boldsymbol{T}^{+}\boldsymbol{V}'+\boldsymbol{C}\boldsymbol{X}\boldsymbol{Q}\boldsymbol{X}'_{0} $$

is symmetric. From (4), condition (2) is proved.

(iii) With the expression of ***G***, it is obviously seen that
  6 $$ \mathscr{M}(\boldsymbol{G})\subseteq\mathscr{M}(\boldsymbol{C}\boldsymbol{X}-\boldsymbol{X}_{0}). $$

If $\boldsymbol{C}\boldsymbol{X}=\boldsymbol{X}_{0}$, $\mathscr{M}(\boldsymbol{C}\boldsymbol{X}-\boldsymbol{X}_{0})\subseteq \mathscr{M}(\boldsymbol{G})$. If $\boldsymbol{C}\boldsymbol{X}\neq \boldsymbol{X}_{0}$, we also use reduction to absurdity and suppose that, for any nonzero vector ***η***, $\boldsymbol{\eta}'\boldsymbol{G}\boldsymbol{\eta}=0$, namely

$$\boldsymbol{\eta}'(\boldsymbol{C}\boldsymbol{X}-\boldsymbol{X}_{0})\boldsymbol{Q}(\boldsymbol{C}\boldsymbol{X}-\boldsymbol{X}_{0})'\boldsymbol{\eta}+\boldsymbol{\eta}'(\boldsymbol{C}\boldsymbol{X}-\boldsymbol{X}_{0})\bigl[{\lambda}\bigl(\boldsymbol{X}'\boldsymbol{T}^{+}\boldsymbol{X}\bigr)^{-}\boldsymbol{X}'\boldsymbol{T}^{+}\boldsymbol{V}'-\boldsymbol{Q}\boldsymbol{X}'\boldsymbol{C}'\bigr]\boldsymbol{\eta}=0. $$

Since $(\boldsymbol{C}\boldsymbol{X}-\boldsymbol{X}_{0})[{\lambda}(\boldsymbol{X}'\boldsymbol{T}^{+}\boldsymbol{X})^{-}\boldsymbol{X}'\boldsymbol{T}^{+}\boldsymbol{V}'-\boldsymbol{Q}\boldsymbol{X}'\boldsymbol{C}']$ is symmetric and nonnegative definite by the proof of condition (2), $\boldsymbol{G}\geq\mathbf{0}$ and

$$\begin{aligned} &\boldsymbol{\eta}'(\boldsymbol{C}\boldsymbol{X}-\boldsymbol{X}_{0})\boldsymbol{Q}(\boldsymbol{C}\boldsymbol{X}-\boldsymbol{X}_{0})'\boldsymbol{\eta}=0, \\ &\boldsymbol{\eta}'(\boldsymbol{C}\boldsymbol{X}-\boldsymbol{X}_{0})\bigl[{\lambda}\bigl(\boldsymbol{X}'\boldsymbol{T}^{+}\boldsymbol{X}\bigr)^{-}\boldsymbol{X}'\boldsymbol{T}^{+}\boldsymbol{V}'-\boldsymbol{Q}\boldsymbol{X}'\boldsymbol{C}'\bigr]\boldsymbol{\eta}=0, \end{aligned}$$

which are equivalent to

7 $$\begin{aligned}& \begin{aligned}&\boldsymbol{\eta}'(\boldsymbol{C}\boldsymbol{X}-\boldsymbol{X}_{0})\boldsymbol{Q}=\mathbf{0}, \\ &\boldsymbol{\eta}'(\boldsymbol{C}\boldsymbol{X}-\boldsymbol{X}_{0})[\lambda(\boldsymbol{X}'\boldsymbol{T}^{+}\boldsymbol{X})^{-}\boldsymbol{X}'\boldsymbol{T}^{+}\boldsymbol{V}']= \mathbf{0}. \end{aligned} \end{aligned}$$

Let $\boldsymbol{\xi}'\boldsymbol{y}$ be a predictor of $\boldsymbol{\eta}'\boldsymbol{y}_{0}$, where

$$\boldsymbol{\xi}'=\boldsymbol{\eta}'\bigl\{ \boldsymbol{X}_{0}\bigl(\boldsymbol{X}'\boldsymbol{T}^{+}\boldsymbol{X}\bigr)^{-}\boldsymbol{X}'\boldsymbol{T}^{+} +{\lambda} \boldsymbol{V}\boldsymbol{T}^{+}\bigl[\boldsymbol{I}-\boldsymbol{X}\bigl(\boldsymbol{X}'\boldsymbol{T}^{+}\boldsymbol{X}\bigr)^{-}\boldsymbol{X}'\boldsymbol{T}^{+}\bigr] \bigr\} . $$

It can be verified by Lemma 2.1 that $\text{R}(\boldsymbol{\xi}'\boldsymbol{y};\boldsymbol{\beta})\geq \text{R}(\boldsymbol{\eta}'C\boldsymbol{y};\boldsymbol{\beta})$ since $\boldsymbol{\eta}'\boldsymbol{C}\boldsymbol{y}\sim \boldsymbol{\eta}'\boldsymbol{y}_{0}$. From Lemma 2.4 and (7), we obtain

$$\begin{aligned} \text{R}\bigl(\boldsymbol{\eta}'\boldsymbol{C}\boldsymbol{y};\boldsymbol{\beta}\bigr)-\text{R}\bigl(\boldsymbol{\xi}'\boldsymbol{y}; \boldsymbol{\beta}\bigr) ={}&\boldsymbol{\eta}'\bigl\{ \boldsymbol{C}\boldsymbol{X}\boldsymbol{Q}\boldsymbol{X}'\boldsymbol{C}'-\boldsymbol{X}_{0}\boldsymbol{Q}\boldsymbol{X}_{0}'-2{\lambda}(\boldsymbol{C}\boldsymbol{X}-\boldsymbol{X}_{0}) \bigl(\boldsymbol{X}'\boldsymbol{T}^{+}\boldsymbol{X}\bigr)^{-}\boldsymbol{X}'\boldsymbol{T}^{+}\boldsymbol{V}\\ &{}+\boldsymbol{\beta}'(\boldsymbol{C}\boldsymbol{X}-\boldsymbol{X}_{0})'(\boldsymbol{C}\boldsymbol{X}-\boldsymbol{X}_{0})\boldsymbol{\beta}\bigr\} \boldsymbol{\eta}\\ ={}&\boldsymbol{\eta}'\boldsymbol{\beta}'(\boldsymbol{C}\boldsymbol{X}-\boldsymbol{X}_{0})'( \boldsymbol{C}\boldsymbol{X}-\boldsymbol{X}_{0})\boldsymbol{\beta} \boldsymbol{\eta}\geq0. \end{aligned}$$

As $\boldsymbol{C}\boldsymbol{X}\neq \boldsymbol{X}_{0}$, there at least exists one $\boldsymbol{\beta}_{0}\in \mathbb{R}^{p}$ such that $\text{R}(\boldsymbol{\eta}'\boldsymbol{C}\boldsymbol{y};\boldsymbol{\beta}_{0})-\text{R}(\boldsymbol{\xi}'\boldsymbol{y};\boldsymbol{\beta}_{0})=\boldsymbol{\eta}'\boldsymbol{\beta}_{0}'(\boldsymbol{C}\boldsymbol{X}-\boldsymbol{X}_{0})'(\boldsymbol{C}\boldsymbol{X}-\boldsymbol{X}_{0})\boldsymbol{\beta}_{0}\boldsymbol{\eta}>0$, which is contradicted to the admissibility of $\boldsymbol{\eta}'C\boldsymbol{y}$. Therefore, if $\boldsymbol{C}\boldsymbol{X}\neq \boldsymbol{X}_{0}$, then $\boldsymbol{\eta}'\boldsymbol{G}\boldsymbol{\eta}\neq0$. Equivalently, if $\boldsymbol{\eta}'G\boldsymbol{\eta}= 0$, then $\boldsymbol{C}\boldsymbol{X}= \boldsymbol{X}_{0}$. Because $\boldsymbol{\eta}'\boldsymbol{G}\boldsymbol{\eta}= 0\Leftrightarrow \boldsymbol{\eta}'\boldsymbol{G}=\mathbf{0}$, then if $\boldsymbol{\eta}'\boldsymbol{G}=\mathbf{0} $, $\boldsymbol{\eta}'(\boldsymbol{C}\boldsymbol{X}-\boldsymbol{X}_{0})=\mathbf{0}$, namely $\mathscr{M}(\boldsymbol{C}\boldsymbol{X}-\boldsymbol{X}_{0})\subseteq\mathscr{M}(\boldsymbol{G})$. Together with (6), $\mathscr{M}(\boldsymbol{C}\boldsymbol{X}-\boldsymbol{X}_{0})=\mathscr{M}(\boldsymbol{G})$.

Sufficiency: Let ***My*** be an arbitrary predictor of $\boldsymbol{y}_{0}$, by Lemma 2.3, we only need to prove that if $\boldsymbol{M}\boldsymbol{\Sigma}=\widetilde{\boldsymbol{M}}\boldsymbol{\Sigma}$, where $\widetilde{\boldsymbol{M}}=\boldsymbol{M}\boldsymbol{X}(\boldsymbol{X}'\boldsymbol{T}^{+}\boldsymbol{X})^{-}\boldsymbol{X}'\boldsymbol{T}^{+}+{\lambda} \boldsymbol{V}\boldsymbol{T}^{+}[\boldsymbol{I}-\boldsymbol{X}(\boldsymbol{X}'\boldsymbol{T}^{+}\boldsymbol{X})^{-}\boldsymbol{X}'\boldsymbol{T}^{+}]$, then ***My*** cannot be better than ***Cy***. Or, we can only prove that there exists at least one $\boldsymbol{\beta}^{*}$ such that $\text{R}(\boldsymbol{M}\boldsymbol{y};\boldsymbol{\beta}^{*})>\text{R}(\boldsymbol{C}\boldsymbol{y};\boldsymbol{\beta}^{*})$. We divide the issue into two circumstances:

(i) $\boldsymbol{C}\boldsymbol{X}=\boldsymbol{X}_{0}$:
  If $\boldsymbol{M}\boldsymbol{X}\neq \boldsymbol{X}_{0}$, it is obviously that ***My*** cannot be better than ***Cy*** by computing their risks;
  If $\boldsymbol{M}\boldsymbol{X}=\boldsymbol{X}_{0}$, $\text{R}(\boldsymbol{M}\boldsymbol{y};\boldsymbol{\beta})=\text{R}(\boldsymbol{C}\boldsymbol{y};\boldsymbol{\beta})$.
(ii) $\boldsymbol{C}\boldsymbol{X}\neq \boldsymbol{X}_{0}$. There are two cases to be discussed:
  - $\boldsymbol{M}\boldsymbol{X}=\boldsymbol{X}_{0}$. By condition (2),
    $$\begin{aligned}& \text{R}(\boldsymbol{M}\boldsymbol{y};\mathbf{0})-\text{R}(\boldsymbol{C}\boldsymbol{y};\mathbf{0})-\operatorname{tr}(\boldsymbol{G}) \\& \quad=\operatorname{tr}\bigl[\boldsymbol{X}_{0}\boldsymbol{Q}\boldsymbol{X}_{0}'-\boldsymbol{C}\boldsymbol{X}\boldsymbol{Q}\boldsymbol{X}_{0}'+{\lambda}(\boldsymbol{C}\boldsymbol{X}-\boldsymbol{X}_{0}) \bigl(\boldsymbol{X}'\boldsymbol{T}^{+}\boldsymbol{X}\bigr)^{-}\boldsymbol{X}'\boldsymbol{T}^{+}\boldsymbol{V}'\bigr] \\& \quad\geq \operatorname{tr}\bigl(\boldsymbol{X}_{0}\boldsymbol{Q}\boldsymbol{X}_{0}'-\boldsymbol{C}\boldsymbol{X}\boldsymbol{Q}\boldsymbol{X}_{0}'+\boldsymbol{C}\boldsymbol{X}\boldsymbol{Q}\boldsymbol{X}'\boldsymbol{C}'-\boldsymbol{C}\boldsymbol{X}\boldsymbol{Q}\boldsymbol{X}_{0}'\bigr) \\& \quad=\operatorname{tr}\bigl[(\boldsymbol{C}\boldsymbol{X}-\boldsymbol{X}_{0})\boldsymbol{Q}(\boldsymbol{C}\boldsymbol{X}-\boldsymbol{X}_{0})'\bigr]\geq0. \end{aligned}$$
    Since $\boldsymbol{C}\boldsymbol{X}\neq \boldsymbol{X}_{0}$ and by condition (3), $\operatorname{rk}(\boldsymbol{G})=\operatorname{rk}(\boldsymbol{C}\boldsymbol{X}-\boldsymbol{X}_{0})\geq1$, and $\operatorname{tr}(\boldsymbol{G})>0$. Thus, $\text{R}(\boldsymbol{M}\boldsymbol{y};0)>\text{R}(\boldsymbol{C}\boldsymbol{y};0)$ and ***My*** cannot be better than ***Cy***.
  - $\boldsymbol{M}\boldsymbol{X}\neq \boldsymbol{X}_{0}$. If $\boldsymbol{M}\boldsymbol{X}=\boldsymbol{C}\boldsymbol{X}$, then $\text{R}(\boldsymbol{M}\boldsymbol{y};\boldsymbol{\beta})=\text{R}(\boldsymbol{C}\boldsymbol{y};\boldsymbol{\beta})$. So we only need to discuss this issue on the case that $\boldsymbol{M}\boldsymbol{X}\neq \boldsymbol{C}\boldsymbol{X}$. By Lemma 2.4 and for any ***β***, we only need to consider the admissibility under this circumstance that
    $$\begin{aligned} \boldsymbol{\beta}'(\boldsymbol{M}\boldsymbol{X}-\boldsymbol{X}_{0})'(\boldsymbol{M}\boldsymbol{X}-\boldsymbol{X}_{0})\boldsymbol{\beta}\leq \boldsymbol{\beta}'(\boldsymbol{C}\boldsymbol{X}-\boldsymbol{X}_{0})'( \boldsymbol{C}\boldsymbol{X}-\boldsymbol{X}_{0})\boldsymbol{\beta}, \end{aligned}$$
    namely
    8 $$\begin{aligned} (\boldsymbol{M}\boldsymbol{X}-\boldsymbol{X}_{0})'(\boldsymbol{M}\boldsymbol{X}-\boldsymbol{X}_{0}) \leq(\boldsymbol{C}\boldsymbol{X}-\boldsymbol{X}_{0})'(\boldsymbol{C}\boldsymbol{X}-\boldsymbol{X}_{0}). \end{aligned}$$
    It can be shown from (6) that $\mathscr{M}[(\boldsymbol{M}\boldsymbol{X}-\boldsymbol{X}_{0})'] \subset\mathscr{M}[(\boldsymbol{C}\boldsymbol{X}-\boldsymbol{X}_{0})']$, then
    9 $$\begin{aligned} (\boldsymbol{M}\boldsymbol{X}-\boldsymbol{X}_{0})'=(\boldsymbol{C}\boldsymbol{X}-\boldsymbol{X}_{0})' \boldsymbol{Z}, \end{aligned}$$
    where
    $$\boldsymbol{Z}=\bigl[(\boldsymbol{C}\boldsymbol{X}-\boldsymbol{X}_{0}) (\boldsymbol{C}\boldsymbol{X}-\boldsymbol{X}_{0})' \bigr]^{+}(\boldsymbol{C}\boldsymbol{X}-\boldsymbol{X}_{0}) (\boldsymbol{M}\boldsymbol{X}-\boldsymbol{X}_{0})'. $$
    By (8), $\boldsymbol{Z}\boldsymbol{Z}'\leq[(\boldsymbol{C}\boldsymbol{X}-\boldsymbol{X}_{0})(\boldsymbol{C}\boldsymbol{X}-\boldsymbol{X}_{0})']^{+}(\boldsymbol{C}\boldsymbol{X}-\boldsymbol{X}_{0})(\boldsymbol{C}\boldsymbol{X}-\boldsymbol{X}_{0})'\leq \boldsymbol{I}$. By (9) and condition (2), we have
    $$\begin{aligned} &\text{R}(\boldsymbol{M}\boldsymbol{y};\mathbf{0})-\text{R}(\boldsymbol{C}\boldsymbol{y};\mathbf{0}) \\ &\quad=\operatorname{tr}\bigl[(\boldsymbol{M}\boldsymbol{X}-\boldsymbol{X}_{0})\boldsymbol{Q}(\boldsymbol{M}\boldsymbol{X}-\boldsymbol{X}_{0})'-2 {\lambda}(\boldsymbol{M}\boldsymbol{X}-\boldsymbol{X}_{0})\boldsymbol{X}'\boldsymbol{T}^{+}\boldsymbol{X})^{-}\boldsymbol{X}'\boldsymbol{T}^{+}\boldsymbol{V}' \\ &\qquad{}+2\boldsymbol{X}_{0}\boldsymbol{Q}(\boldsymbol{M}\boldsymbol{X}-\boldsymbol{X}_{0})'-(\boldsymbol{C}\boldsymbol{X}-\boldsymbol{X}_{0})\boldsymbol{Q}(\boldsymbol{C}\boldsymbol{X}-\boldsymbol{X}_{0})' \\ &\qquad{} +2{\lambda}(\boldsymbol{C}\boldsymbol{X}-\boldsymbol{X}_{0})\boldsymbol{X}'\boldsymbol{T}^{+}\boldsymbol{X})^{-}\boldsymbol{X}'\boldsymbol{T}^{+}\boldsymbol{V}'-2\boldsymbol{X}_{0}\boldsymbol{Q}(\boldsymbol{C}\boldsymbol{X}-\boldsymbol{X}_{0})'\bigr] \\ &\quad= \operatorname{tr}\bigl[(\boldsymbol{I}-\boldsymbol{Z}) (\boldsymbol{I}-\boldsymbol{Z})'\boldsymbol{X}_{0}\boldsymbol{Q}\boldsymbol{X}_{0}'+2\boldsymbol{Z}(\boldsymbol{I}-\boldsymbol{Z})'\boldsymbol{X}_{0}\boldsymbol{Q}\boldsymbol{X}'\boldsymbol{C}' \\ &\qquad{}+2{\lambda}(\boldsymbol{I}-\boldsymbol{Z})'(\boldsymbol{C}\boldsymbol{X}-\boldsymbol{X}_{0}) \bigl(\boldsymbol{X}'\boldsymbol{T}^{+}\boldsymbol{X}\bigr)^{-}\boldsymbol{X}'\boldsymbol{T}^{+}\boldsymbol{V}'-\bigl(\boldsymbol{I}-\boldsymbol{Z}\boldsymbol{Z}'\bigr)\boldsymbol{C}\boldsymbol{X}\boldsymbol{Q}\boldsymbol{X}'\boldsymbol{C}'\bigr] \\ &\quad\geq \operatorname{tr}\bigl[(\boldsymbol{I}-\boldsymbol{Z}) (\boldsymbol{I}-\boldsymbol{Z})'\boldsymbol{X}_{0}\boldsymbol{Q}\boldsymbol{X}_{0}'+2\boldsymbol{Z}(\boldsymbol{I}-\boldsymbol{Z})'\boldsymbol{X}_{0}\boldsymbol{Q}\boldsymbol{X}'\boldsymbol{C}' \\ &\qquad{}+2{\lambda}(\boldsymbol{I}-\boldsymbol{Z})'(\boldsymbol{C}\boldsymbol{X}-\boldsymbol{X}_{0}) \bigl(\boldsymbol{X}'\boldsymbol{T}^{+}\boldsymbol{X}\bigr)^{-}\boldsymbol{X}'\boldsymbol{T}^{+}\boldsymbol{V}' \\ &\qquad{}-{\lambda}\bigl(\boldsymbol{I}-\boldsymbol{Z}\boldsymbol{Z}'\bigr) (\boldsymbol{C}\boldsymbol{X}-\boldsymbol{X}_{0}) \bigl(\boldsymbol{X}'\boldsymbol{T}^{+}\boldsymbol{X}\bigr)^{-}\boldsymbol{X}'\boldsymbol{T}^{+}\boldsymbol{V}'-\bigl(\boldsymbol{I}-\boldsymbol{Z}\boldsymbol{Z}'\bigr)\boldsymbol{X}_{0}\boldsymbol{Q}\boldsymbol{X}'\boldsymbol{C}'\bigr] \\ &\quad= \operatorname{tr}\bigl[(\boldsymbol{I}-\boldsymbol{Z}) (\boldsymbol{I}-\boldsymbol{Z})'\boldsymbol{X}_{0}\boldsymbol{Q}\boldsymbol{X}_{0}'-2(\boldsymbol{I}-\boldsymbol{Z}) \bigl(\boldsymbol{I}-\boldsymbol{Z}'\bigr)\boldsymbol{X}_{0}\boldsymbol{Q}\boldsymbol{X}'\boldsymbol{C}' \\ &\qquad{}+{\lambda}\bigl(\boldsymbol{I}-2\boldsymbol{Z}'+\boldsymbol{Z}\boldsymbol{Z}'\bigr) (\boldsymbol{C}\boldsymbol{X}-\boldsymbol{X}_{0}) \bigl(\boldsymbol{X}'\boldsymbol{T}^{+}\boldsymbol{X}\bigr)^{-} \boldsymbol{X}'\boldsymbol{T}^{+}\boldsymbol{V}'+\boldsymbol{X}_{0}\boldsymbol{Q}\boldsymbol{X}'\boldsymbol{C}'\bigr] \\ &\quad= \operatorname{tr}\bigl[(\boldsymbol{I}-\boldsymbol{Z}) (\boldsymbol{I}-\boldsymbol{Z})'\boldsymbol{X}_{0}\boldsymbol{Q}\boldsymbol{X}_{0}'-2(\boldsymbol{I}-\boldsymbol{Z}) \bigl(\boldsymbol{I}-\boldsymbol{Z}'\bigr)\boldsymbol{X}_{0}\boldsymbol{Q}\boldsymbol{X}'\boldsymbol{C}' \\ &\qquad{}+{\lambda}\bigl(\boldsymbol{I}-\boldsymbol{Z}-\boldsymbol{Z}'+\boldsymbol{Z}\boldsymbol{Z}'\bigr) (\boldsymbol{C}\boldsymbol{X}-\boldsymbol{X}_{0}) \bigl(\boldsymbol{X}'\boldsymbol{T}^{+}\boldsymbol{X}\bigr)^{-} \boldsymbol{X}'\boldsymbol{T}^{+}\boldsymbol{V}'+\boldsymbol{X}_{0}\boldsymbol{Q}\boldsymbol{X}'\boldsymbol{C}'\bigr] \\ &\quad= \operatorname{tr}\bigl[(\boldsymbol{I}-\boldsymbol{Z})'\boldsymbol{G}(\boldsymbol{I}-\boldsymbol{Z})\bigr]. \end{aligned}$$
    Notice that $(\boldsymbol{C}\boldsymbol{X}-\boldsymbol{X}_{0})'(\boldsymbol{I}-\boldsymbol{Z})=(\boldsymbol{C}\boldsymbol{X}-\boldsymbol{X}_{0})'-(\boldsymbol{M}\boldsymbol{X}-\boldsymbol{X}_{0})'=\boldsymbol{C}\boldsymbol{X}-\boldsymbol{M}\boldsymbol{X}\neq\mathbf{0}$ and together with condition (3), $\boldsymbol{G}(\boldsymbol{I}-\boldsymbol{Z})\neq\mathbf{0}$. Moreover, as $\boldsymbol{G}\geq\mathbf{0}$, then
    $$\text{R}(\boldsymbol{M}\boldsymbol{y};\mathbf{0})-\text{R}(\boldsymbol{C}\boldsymbol{y};\mathbf{0})\geq \operatorname{tr}\bigl[(\boldsymbol{I}-\boldsymbol{Z})'\boldsymbol{G}(\boldsymbol{I}-\boldsymbol{Z})\bigr]>0, $$
    which means ***My*** cannot be better than ***Cy***.

In summary, the proof is complected. □

### Corollary 3.1

*For the model* (3), *if* $\boldsymbol{\Sigma}> \mathbf{0}$, *then* $\boldsymbol{C}\boldsymbol{y}\sim \boldsymbol{\delta}$ *under the quadratic loss function if and only if*

1. $\boldsymbol{C}=\boldsymbol{C}\boldsymbol{X}(\boldsymbol{X}'\boldsymbol{\Sigma}^{-1}\boldsymbol{X})^{-}\boldsymbol{X}'\boldsymbol{\Sigma}^{-1}+{\lambda} \boldsymbol{V}\boldsymbol{\Sigma}^{-1}[\boldsymbol{I}-\boldsymbol{X}(\boldsymbol{X}'\boldsymbol{\Sigma}^{-1}\boldsymbol{X})^{-}\boldsymbol{X}'\boldsymbol{\Sigma}^{-1}]$, *and*
2. $\boldsymbol{C}\boldsymbol{X}\boldsymbol{Q}\boldsymbol{X}'\boldsymbol{C}'\leq {\lambda}(\boldsymbol{C}\boldsymbol{X}-\boldsymbol{X}_{0})(\boldsymbol{X}'\boldsymbol{\Sigma}^{-1}\boldsymbol{X})^{-}\boldsymbol{X}'\boldsymbol{\Sigma}^{-1}\boldsymbol{V}'+\boldsymbol{C}\boldsymbol{X}\boldsymbol{Q}\boldsymbol{X}'_{0}$.

### Proof

If $\boldsymbol{\Sigma}> \mathbf{0}$, then $T> \mathbf{0}$ and

10 $$ \begin{aligned} &\boldsymbol{T}^{+}=\boldsymbol{T}^{-1}= \boldsymbol{\Sigma}^{-1}-\boldsymbol{\Sigma}^{-1}\boldsymbol{X}\bigl(\boldsymbol{I}+\boldsymbol{X}' \boldsymbol{\Sigma}^{-1}\boldsymbol{X}\bigr)^{-1}\boldsymbol{X}'\boldsymbol{\Sigma}^{-1}, \\ &\boldsymbol{X}'\boldsymbol{\Sigma}^{-1}\boldsymbol{X}\bigl(\boldsymbol{I}+\boldsymbol{X}'\boldsymbol{\Sigma}^{-1}\boldsymbol{X}\bigr)^{-1}\boldsymbol{X}'\boldsymbol{\Sigma}^{-1}= \boldsymbol{I}-\bigl(\boldsymbol{I}+\boldsymbol{X}'\boldsymbol{\Sigma}^{-1}\boldsymbol{X}\bigr)^{-1}\boldsymbol{X}'\boldsymbol{\Sigma}^{-1}. \end{aligned} $$

Therefore,

11 $$ \boldsymbol{X}'\boldsymbol{T}^{-1}=\bigl(\boldsymbol{I}+\boldsymbol{X}' \boldsymbol{\Sigma}^{-1}\boldsymbol{X}\bigr)^{-1}\boldsymbol{X}' \Sigma^{-1} $$

and

12 $$\begin{aligned} \boldsymbol{X}\bigl(\boldsymbol{X}'\boldsymbol{T}^{-1}\boldsymbol{X}\bigr)^{-}\boldsymbol{X}'\boldsymbol{T}^{-1}& =\boldsymbol{X}\bigl[\bigl(\boldsymbol{I}+\boldsymbol{X}'\boldsymbol{\Sigma}^{-1}\boldsymbol{X}\bigr)^{-1}\boldsymbol{X}' \boldsymbol{\Sigma}^{-1}\boldsymbol{X}\bigr]^{-}\bigl(I+X'\boldsymbol{\Sigma}^{-1}\boldsymbol{X}\bigr)^{-1}\boldsymbol{X}'\boldsymbol{\Sigma}^{-1} \\ & =\boldsymbol{X}\bigl(\boldsymbol{X}'\boldsymbol{\Sigma}^{-1}\boldsymbol{X}\bigr)^{-}\boldsymbol{X}'\boldsymbol{\Sigma}^{-1}. \end{aligned}$$

By (10), (11) and (12), conditions (1), (2) in Corollary 3.1 hold and condition (3) in Theorem 3.2 holds naturally since $\operatorname{rk}(\boldsymbol{C}\boldsymbol{X}-\boldsymbol{X}_{0})=\operatorname{rk}(\boldsymbol{G})$. □

### Remark 3.1

As $\boldsymbol{\delta}=\boldsymbol{y}_{0}$ when ${\lambda}=1$ and $\boldsymbol{\delta}=\text{E}\boldsymbol{y}_{0}$ when ${\lambda}=0$, it is convenient to obtain the sufficient and necessary conditions for the predictors of $\boldsymbol{y}_{0}$ and $\text{E}\boldsymbol{y}_{0}$ to be admissible from Theorem 3.2 and Corollary 3.1.

### Corollary 3.2

*For model* (3) *and under the quadratic loss function*, *the best linear unbiased predictors of* ***δ***, $\boldsymbol{y}_{0}$ *and* $\text{E}\boldsymbol{y}_{0}$ *are*

$$\begin{aligned} &\hat{\boldsymbol{\delta}}_{{\mathrm{BLUP}}}=\boldsymbol{C}\boldsymbol{y}=\boldsymbol{X}_{0}\bigl(\boldsymbol{X}' \boldsymbol{T}^{+}\boldsymbol{X}\bigr)^{-}\boldsymbol{X}'\boldsymbol{T}^{+}\boldsymbol{y}+{\lambda} \boldsymbol{V}\boldsymbol{T}^{+}\bigl[\boldsymbol{y}-\boldsymbol{X}\bigl(\boldsymbol{X}'\boldsymbol{T}^{+}\boldsymbol{X}\bigr)^{-}\boldsymbol{X}'\boldsymbol{T}^{+}\boldsymbol{y}\bigr], \\ &\hat{\boldsymbol{y}}_{0_{{\mathrm{BLUP}}}}=\boldsymbol{X}_{0}\bigl(\boldsymbol{X}'\boldsymbol{T}^{+}\boldsymbol{X}\bigr)^{-}\boldsymbol{X}'\boldsymbol{T}^{+}\boldsymbol{y}+ \boldsymbol{V}\boldsymbol{T}^{+}\bigl[\boldsymbol{y}-\boldsymbol{X}\bigl(\boldsymbol{X}'\boldsymbol{T}^{+}\boldsymbol{X}\bigr)^{-}\boldsymbol{X}'\boldsymbol{T}^{+}\boldsymbol{y}\bigr], \\ &\hat{\text{E}}\boldsymbol{y}_{0_{{\mathrm{BLUP}}}}=\boldsymbol{X}_{0}\bigl(\boldsymbol{X}'\boldsymbol{T}^{+}\boldsymbol{X}\bigr)^{-}\boldsymbol{X}'\boldsymbol{T}^{+}\boldsymbol{y}. \end{aligned}$$

### Proof

Let $\boldsymbol{C}\boldsymbol{y}+\boldsymbol{u}$ be the linear unbiased predictor of ***δ***. $\text{E}(\boldsymbol{C}\boldsymbol{y}+\boldsymbol{u})=\text{E}\boldsymbol{\delta}$ gives that $\boldsymbol{C}\boldsymbol{X}=\boldsymbol{X}_{0}$ and $\boldsymbol{u}=\mathbf{0}$. Let $\boldsymbol{C}=\boldsymbol{X}_{0}(\boldsymbol{X}'\boldsymbol{T}^{+}\boldsymbol{X})^{-}\boldsymbol{X}'\boldsymbol{T}^{+}+{\lambda} \boldsymbol{V}\boldsymbol{T}^{+}[\boldsymbol{I}-\boldsymbol{X}(\boldsymbol{X}'\boldsymbol{T}^{+}\boldsymbol{X})^{-}\boldsymbol{X}'\boldsymbol{T}^{+}]$, the corollary is easily proved by verifying the conditions in Theorem 3.2. □

We give some admissible predictors in the following examples.

### Example 3.1

Suppose $\boldsymbol{\Sigma}>\mathbf{0}$, the best linear unbiased predictor of $\boldsymbol{y}_{0}$ in [1] is $\hat{\boldsymbol{y}} _{0_{\mathrm{BLUP}}}=\boldsymbol{X}_{0}(\boldsymbol{X}'\boldsymbol{\Sigma}^{-1}\boldsymbol{X})^{-}\boldsymbol{X}'\boldsymbol{\Sigma}^{-1}\boldsymbol{y}+\boldsymbol{V}\boldsymbol{\Sigma}^{-1}[\boldsymbol{I}-\boldsymbol{X}(\boldsymbol{X}'\boldsymbol{\Sigma}^{-1}\boldsymbol{X})^{-}\boldsymbol{X}'\boldsymbol{\Sigma}^{-1}]\boldsymbol{y}$. Let $\hat{\boldsymbol{\beta}}_{{\mathrm{BLUE}}}=(\boldsymbol{X}'\boldsymbol{\Sigma}^{-1}\boldsymbol{X})^{-1}\boldsymbol{X}'\boldsymbol{\Sigma}^{-1}\boldsymbol{y}$. $\hat{\boldsymbol{y}}_{0_{\mathrm{SPP}}}=\boldsymbol{X}_{0}\hat{\boldsymbol{\beta}}_{{\mathrm{BLUE}}}$ is the simple projective predictor of $\boldsymbol{y}_{0}$ in [6]. Note that $\text{E}\hat{\boldsymbol{y}}_{0_{\mathrm{BLUP}}}=\text{E}\boldsymbol{\delta}= \text{E}\boldsymbol{y}_{0_{\mathrm{SPP}}}$, which means $\hat{\boldsymbol{y}}_{0_{\mathrm{BLUP}}}$ and $\hat{\boldsymbol{y}}_{0_{\mathrm{SPP}}}$ are also unbiased predictors of ***δ***. If ${\lambda}=1$, then by Corollary 3.1, $\hat{\boldsymbol{y}}_{0_{\mathrm{BLUP}}}\sim \boldsymbol{y}_{0}$, and therefore $\hat{\boldsymbol{y}}_{0_{\mathrm{BLUP}}} \nsim \boldsymbol{\delta}$ if ${\lambda}\neq1$. If ${\lambda}=0$, then by Corollary 3.1, $\hat{\boldsymbol{y}}_{0_{\mathrm{SPP}}}\sim \text{E}\boldsymbol{y}_{0}$, which means if ${\lambda}\neq0$, $\hat{\boldsymbol{y}}_{0_{\mathrm{SPP}}}\nsim \boldsymbol{\delta}$.

### Remark 3.2

Example 3.1 indicates that the unbiased predictors of ***δ*** are also the unbiased predictors of $\boldsymbol{y}_{0}$ and $\text{E}\boldsymbol{y}_{0}$, and the unbiased predictors of $\boldsymbol{y}_{0}$ and $\text{E}\boldsymbol{y}_{0}$ are also the unbiased predictors of ***δ*** since $\text{E}\boldsymbol{\delta}=\text{E}\boldsymbol{y}_{0}=\text{E}(\text{E}\boldsymbol{y}_{0})=\boldsymbol{X}_{0}\boldsymbol{\beta}$. However, admissibility of those predictors for each studied variables are different.

### Example 3.2

Suppose $\boldsymbol{V}=\mathbf{0}$, $\Sigma>\mathbf{0}$ and $\operatorname{rk}(\boldsymbol{X})< p$ in model (3). Suppose $t_{{\max}}$ be the maximum eigenvalue of $\boldsymbol{X}'\boldsymbol{X}$, non-stochastic scalars $k>0$ and $\theta =\frac{t _{{\max}}+k}{t_{{\max}}}$. Let

$$\boldsymbol{D}=\boldsymbol{B}-\boldsymbol{B}\bigl[\boldsymbol{I}_{n}-\theta \boldsymbol{X}\bigl(\boldsymbol{X}'\boldsymbol{X}+k\boldsymbol{I}_{p}\bigr)^{-1}\boldsymbol{X}'\bigr]^{-}\bigl[\boldsymbol{I}_{n}-\theta \boldsymbol{X}\bigl(\boldsymbol{X}'\boldsymbol{X}+k\boldsymbol{I}_{p} \bigr)^{-1}X'\bigr], $$

where ***B*** is an $m\times n$ arbitrary matrix. If $\boldsymbol{X}_{0}=\theta \boldsymbol{D}\boldsymbol{X}$, then by Corollary 3.1 without tedious calculations, $\boldsymbol{X}_{0}(\boldsymbol{X}'\boldsymbol{X}+k\boldsymbol{I}_{p})^{-1}\boldsymbol{X}'\boldsymbol{y}\sim \boldsymbol{\delta}$.

### Remark 3.3

Denote $\hat{\boldsymbol{\beta}}_{{\mathrm{ridge}}}$ as the ridge estimator of ***β*** in model (1) when $\boldsymbol{\Sigma}> \mathbf{0}$ and $\operatorname{rk}(\boldsymbol{X})< p$. Example 3.2 indicates that in particular linear regression model, if $\boldsymbol{X}_{0}$ and ***X*** have some relations, we can use the ridge predictor $\boldsymbol{X}_{0}\hat{\boldsymbol{\beta}}_{{\mathrm{ridge}}}$ as the admissible predictor for ***δ***, especially for $\boldsymbol{y}_{0}$ and $\text{E}\boldsymbol{y}_{0}$.

## Admissibility of nonhomogeneous linear predictors

In this section, we investigate the admissibility of simultaneous prediction in class of nonhomogeneous linear predictors, and we obtain the necessary and sufficient conditions. Studies show the admissibility of simultaneous prediction in the class of nonhomogeneous linear predictors is based on the admissibility of simultaneous prediction in the class of homogeneous linear predictors.

### Theorem 4.1

*For the model* (3), $\boldsymbol{C}\boldsymbol{y}+\boldsymbol{u}\sim \boldsymbol{\delta}$ *in* $\mathscr{LN}$ *under the quadratic loss function if and only if*

1. $\boldsymbol{C}\boldsymbol{y}\sim \boldsymbol{\delta}$ *in* $\mathscr{LH}$, *and*
2. $\boldsymbol{u}\in\mathscr{M}(\boldsymbol{C}\boldsymbol{X}-\boldsymbol{X}_{0})$.

### Proof

Necessity: Suppose by contradiction that $\boldsymbol{u}\notin\mathscr{M}(\boldsymbol{C}\boldsymbol{X}-\boldsymbol{X}_{0})$ and $\boldsymbol{P}=(\boldsymbol{C}\boldsymbol{X}-\boldsymbol{X}_{0})[(\boldsymbol{C}\boldsymbol{X}-\boldsymbol{X}_{0})'(\boldsymbol{C}\boldsymbol{X}-\boldsymbol{X}_{0})]^{-}(\boldsymbol{C}\boldsymbol{X}-\boldsymbol{X}_{0})'$, then $(\boldsymbol{I}-\boldsymbol{P})\boldsymbol{u} \neq\mathbf{0}$ and

13 $$\begin{aligned} \text{R}(\boldsymbol{C}\boldsymbol{y}+\boldsymbol{u};\boldsymbol{\beta}) ={}&\text{E}\bigl[ \bigl(\boldsymbol{C}\boldsymbol{y}+\boldsymbol{u}-{\lambda} \boldsymbol{y}_{0}-(1-{\lambda})\boldsymbol{X}_{0}\boldsymbol{\beta}\bigr)'\bigl(\boldsymbol{C}\boldsymbol{y}+ \boldsymbol{u}-{\lambda} \boldsymbol{y}_{0}-(1-{\lambda})\boldsymbol{X}_{0}\boldsymbol{\beta}\bigr) \bigr] \\ ={}&\operatorname{tr}\bigl[\boldsymbol{C}\boldsymbol{\Sigma} \boldsymbol{C}')+{\lambda}^{2} \boldsymbol{\Sigma}_{0}-2{\lambda}\bigl(\boldsymbol{C}\boldsymbol{V}'\bigr)\bigr]+\boldsymbol{\beta}'(\boldsymbol{C}\boldsymbol{X}-\boldsymbol{X}_{0})'(\boldsymbol{C}\boldsymbol{X}-\boldsymbol{X}_{0})\boldsymbol{\beta}\\ &{}+\boldsymbol{u}'\boldsymbol{u}+2\boldsymbol{u}'(\boldsymbol{C}\boldsymbol{X}-\boldsymbol{X}_{0}) \boldsymbol{\beta}\\ ={}&\operatorname{tr}\bigl[\boldsymbol{C}\boldsymbol{\Sigma} \boldsymbol{C}')+{\lambda}^{2} \boldsymbol{\Sigma}_{0}-2{\lambda}\bigl(\boldsymbol{C}\boldsymbol{V}'\bigr)\bigr]+\boldsymbol{\beta}'(\boldsymbol{C}\boldsymbol{X}-\boldsymbol{X}_{0})'(\boldsymbol{C}\boldsymbol{X}-\boldsymbol{X}_{0})\boldsymbol{\beta}\\ &{}+\boldsymbol{u}'\boldsymbol{P}\boldsymbol{u}+\boldsymbol{u}'(\boldsymbol{I}-\boldsymbol{P})\boldsymbol{u}+2\boldsymbol{u} '(\boldsymbol{C}\boldsymbol{X}-\boldsymbol{X}_{0})\boldsymbol{\beta}\\ \geq{}& \operatorname{tr}\bigl[\boldsymbol{C}\boldsymbol{\Sigma} \boldsymbol{C}')+{\lambda}^{2} \boldsymbol{\Sigma}_{0}-2{\lambda}\bigl(\boldsymbol{C}\boldsymbol{V}'\bigr)\bigr]+\boldsymbol{\beta}'(\boldsymbol{C}\boldsymbol{X}-\boldsymbol{X}_{0})'(\boldsymbol{C}\boldsymbol{X}-\boldsymbol{X}_{0})\boldsymbol{\beta}\\ &{}+\boldsymbol{u}'\boldsymbol{P}\boldsymbol{u}+2\boldsymbol{u}'\boldsymbol{P}(\boldsymbol{C}\boldsymbol{X}-\boldsymbol{X}_{0})\boldsymbol{\beta}\\ ={}&\text{R}(\boldsymbol{C}\boldsymbol{y}+\boldsymbol{P}\boldsymbol{u};\beta). \end{aligned}$$

This contradicts the fact that $\boldsymbol{C}\boldsymbol{y}+\boldsymbol{u}\sim\delta$, and therefore $\boldsymbol{u}\in\mathscr{M}(\boldsymbol{C}\boldsymbol{X}-\boldsymbol{X}_{0})$.

Now suppose $\boldsymbol{u}\in\mathscr{M}(\boldsymbol{C}\boldsymbol{X}-\boldsymbol{X}_{0})$ while ***Cy*** is not admissible, then there exists a predictor $\boldsymbol{H}\boldsymbol{y}\in \mathscr{LH}$ of $\boldsymbol{y}_{0}$ such that $\text{R}(H\boldsymbol{y};\boldsymbol{\beta})\leq \text{R}(C\boldsymbol{y};\boldsymbol{\beta})$ for any ***β*** and the inequality holds at least at some $\boldsymbol{\beta}_{0}$. As $\boldsymbol{u}\in\mathscr{M}(\boldsymbol{C}\boldsymbol{X}-\boldsymbol{X}_{0})$, there exists some vector ***p*** such that $\boldsymbol{u}=(\boldsymbol{C}\boldsymbol{X}-\boldsymbol{X}_{0})\boldsymbol{p}$. Then, similar to the derivation of (13),

$$\begin{aligned}& \text{R}\bigl[\boldsymbol{H}\boldsymbol{y}+(\boldsymbol{H}\boldsymbol{X}-\boldsymbol{X}_{0})\boldsymbol {p};\boldsymbol{\beta}\bigr] \\& \quad=\operatorname{tr}\bigl[\boldsymbol{H}\boldsymbol{\Sigma}\boldsymbol{H}')+{\lambda}^{2} \boldsymbol{\Sigma}_{0}-2{\lambda}\bigl(\boldsymbol{H}\boldsymbol{V}'\bigr)\bigr]+\boldsymbol{\beta}'(\boldsymbol{H}\boldsymbol{X}-\boldsymbol{X}_{0})'(\boldsymbol{H}\boldsymbol{X}-\boldsymbol{X}_{0})\boldsymbol{\beta}\\& \qquad{}+\boldsymbol{p}'(\boldsymbol{H}\boldsymbol{X}-\boldsymbol{X}_{0})'(\boldsymbol {H}\boldsymbol{X}-\boldsymbol{X}_{0})\boldsymbol{p}+2\boldsymbol{p}'(\boldsymbol{H}\boldsymbol{X}-\boldsymbol{X}_{0})'( \boldsymbol{H}\boldsymbol{X}-\boldsymbol{X}_{0})\boldsymbol{\beta}\\& \quad=\operatorname{tr}\bigl[\boldsymbol{H}\boldsymbol{\Sigma}\boldsymbol{H}')+{\lambda}^{2} \boldsymbol{\Sigma}_{0}-2{\lambda}\bigl(\boldsymbol{H}\boldsymbol{V}'\bigr)\bigr]+(\boldsymbol{\beta}+ \boldsymbol{p})'(\boldsymbol{H}\boldsymbol{X}-\boldsymbol{X}_{0})'(\boldsymbol{H}\boldsymbol{X}-\boldsymbol{X}_{0}) (\boldsymbol{\beta}+\boldsymbol{p}) \\& \quad=\text{R}(\boldsymbol{H}\boldsymbol{y};\boldsymbol{\beta}+\boldsymbol{p}) \\& \quad\leq \text{R}(\boldsymbol{C}\boldsymbol{y};\boldsymbol{\beta}+\boldsymbol{p}) \\& \quad =\operatorname{tr}\bigl[\boldsymbol{C}\boldsymbol{\Sigma} \boldsymbol{C}')+{\lambda}^{2} \boldsymbol{\Sigma}_{0}-2{\lambda}\bigl(\boldsymbol{C}\boldsymbol{V}'\bigr) \bigr]+(\boldsymbol{\beta}+\boldsymbol{p})'(\boldsymbol{C}\boldsymbol{X}-\boldsymbol{X}_{0})'(\boldsymbol{C}\boldsymbol{X}- \boldsymbol{X}_{0}) (\boldsymbol{\beta}+\boldsymbol{p}) \\& \quad=\text{R}(\boldsymbol{C}\boldsymbol{y}+\boldsymbol{p};\boldsymbol{\beta}) \end{aligned}$$

at any ***β*** and the inequality holds at $\boldsymbol{\beta}=\boldsymbol{\beta}_{0}-\boldsymbol{p}$, which contradicts $\boldsymbol{C}\boldsymbol{y}+\boldsymbol{u}\sim \boldsymbol{\delta}$. Therefore, ***Cy*** is admissible.

Sufficiency: Suppose $\boldsymbol{N}\boldsymbol{y}+\boldsymbol{v}$ is a predictor of $\boldsymbol{y}_{0}$ and for any $\boldsymbol{\beta}\in\mathbb{R}^{\boldsymbol{p}}$, $\text{R}(\boldsymbol{N}\boldsymbol{y}+v;\boldsymbol{\beta})\leq \text{R}(\boldsymbol{C}\boldsymbol{y}+\boldsymbol{u};\boldsymbol{\beta})$. Let $\boldsymbol{P}_{v}$ and $\boldsymbol{P}_{u}$ be the orthogonal projection matrices onto $\mathscr{M}(\boldsymbol{N}\boldsymbol{X}-\boldsymbol{X}_{0})$ and $\mathscr{M}(\boldsymbol{C}\boldsymbol{X}-\boldsymbol{X}_{0})$ respectively. Since $\boldsymbol{u}\in\mathscr{M}(\boldsymbol{C}\boldsymbol{X}-\boldsymbol{X}_{0})$, there exist $\boldsymbol{\beta}_{v}$ and $\boldsymbol{\beta}_{u}$ in $\mathbb{R}^{p}$ such that

$$\begin{aligned}& \boldsymbol{P}_{v}\boldsymbol{v}=(\boldsymbol{N}\boldsymbol{X}-\boldsymbol{X}_{0})\boldsymbol{\beta}_{v}, \\& \boldsymbol {u}=\boldsymbol{P}_{u}\boldsymbol{u}=(\boldsymbol{C}\boldsymbol{X}-\boldsymbol{X}_{0})\boldsymbol{\beta}_{u}. \end{aligned}$$

According to the derivation of (13), we have

14 $$ \text{R}(\boldsymbol{N}\boldsymbol{y}+\boldsymbol{P}_{v}\boldsymbol{v};\boldsymbol{\beta})\leq \text{R}(\boldsymbol{N}\boldsymbol{y}+\boldsymbol {v};\boldsymbol{\beta}) \leq \text{R}(\boldsymbol{C}\boldsymbol{y}+\boldsymbol{u};\boldsymbol{\beta}) =\text{R}(\boldsymbol{C}\boldsymbol{y}+\boldsymbol{P}_{u}\boldsymbol {u};\boldsymbol{\beta}). $$

Equation (14) holds for any ***β*** so that

15 $$\begin{aligned} &(\boldsymbol{N}\boldsymbol{X}-\boldsymbol{X}_{0})'(\boldsymbol{N}\boldsymbol{X}-\boldsymbol{X}_{0})\leq(\boldsymbol{C}\boldsymbol{X}-\boldsymbol{X}_{0})'(\boldsymbol{C}\boldsymbol{X}-\boldsymbol{X}_{0}), \end{aligned}$$

16 $$\begin{aligned} &\text{R}(\boldsymbol{N}\boldsymbol{y}+\boldsymbol{P}_{v}\boldsymbol{v};\boldsymbol{\beta})=\text{R}(\boldsymbol{N}\boldsymbol{y};\boldsymbol{\beta}+\boldsymbol{\beta}_{v})\leq \text{R}(\boldsymbol{C}\boldsymbol{y};\boldsymbol{\beta}+\boldsymbol{\beta}_{u})=\text{R}(\boldsymbol{C}\boldsymbol{y}+\boldsymbol{P}_{u}\boldsymbol{u};\boldsymbol{\beta}). \end{aligned}$$

Let $\boldsymbol{\beta}=-\boldsymbol{\beta}_{u}$ in (16), we have

17 $$\begin{aligned} \text{R}(\boldsymbol{N}\boldsymbol{y};\mathbf{0})\leq \text{R}(\boldsymbol{H}\boldsymbol{y};\boldsymbol{\beta}_{v}-\boldsymbol{\beta}_{u})\leq \text{R}(\boldsymbol{C}\boldsymbol{y}; \mathbf{0}), \end{aligned}$$

and by (15) and (17),

18 $$\begin{aligned} \begin{aligned} \text{R}(\boldsymbol{N}\boldsymbol{y};\boldsymbol{\beta}) ={}&\text{R}(\boldsymbol{N}\boldsymbol{y};\mathbf{0})+\boldsymbol{\beta}'(\boldsymbol{N}\boldsymbol{X}-\boldsymbol{X}_{0})'(\boldsymbol{N}\boldsymbol{X}-\boldsymbol{X}_{0})\boldsymbol{\beta}\\ \leq{}&\text{R}(\boldsymbol{C}\boldsymbol{y};\mathbf{0})+\boldsymbol{\beta}'(\boldsymbol{C}\boldsymbol{X}-\boldsymbol{X}_{0})'( \boldsymbol{C}\boldsymbol{X}-\boldsymbol{X}_{0})\boldsymbol{\beta}=\text{R}(\boldsymbol{C}\boldsymbol{y};\boldsymbol{\beta}). \end{aligned} \end{aligned}$$

Since $\boldsymbol{C}\boldsymbol{y}\sim \boldsymbol{\delta}$ and ***β*** is arbitrary, (18), (17) and (15) successively gives

19 $$\begin{aligned} &\text{R}(\boldsymbol{N}\boldsymbol{y};\boldsymbol{\beta}) =\text{R}(\boldsymbol{C}\boldsymbol{y};\boldsymbol{\beta}), \\ &\text{R}(\boldsymbol{N}\boldsymbol{y};\mathbf{0}) =\text{R}(\boldsymbol{H}\boldsymbol{y};\boldsymbol{\beta}_{v}-\boldsymbol{\beta}_{u})=\text{R}(\boldsymbol{C}\boldsymbol{y}; \mathbf{0}), \end{aligned}$$

20 $$\begin{aligned} &(\boldsymbol{N}\boldsymbol{X}-\boldsymbol{X}_{0})'(\boldsymbol{N}\boldsymbol{X}-\boldsymbol{X}_{0}) = (\boldsymbol{C}\boldsymbol{X}- \boldsymbol{X}_{0})'(\boldsymbol{C}\boldsymbol{X}-\boldsymbol{X}_{0}). \end{aligned}$$

By (19), we have

$$(\boldsymbol{\beta}_{v}-\boldsymbol{\beta}_{u})'(\boldsymbol{N}\boldsymbol{X}-\boldsymbol{X}_{0})'(\boldsymbol{N}\boldsymbol{X}-\boldsymbol{X}_{0}) (\boldsymbol{\beta}_{v}-\boldsymbol{\beta}_{u})=0, $$

namely $(\boldsymbol{N}\boldsymbol{X}-\boldsymbol{X}_{0})(\boldsymbol{\beta}_{v}-\boldsymbol{\beta}_{u})=\mathbf{0}$. In addition, by (20),

$$\begin{aligned} \text{R}(\boldsymbol{N}\boldsymbol{y};\boldsymbol{\beta}+\boldsymbol{\beta}_{v})={}&\text{R}(\boldsymbol{N}\boldsymbol{y};\mathbf{0})+(\boldsymbol{\beta}+ \boldsymbol{\beta}_{v})'(\boldsymbol{N}\boldsymbol{X}-\boldsymbol{X}_{0})'(\boldsymbol{N}\boldsymbol{X}-\boldsymbol{X}_{0}) (\boldsymbol{\beta}+\boldsymbol{\beta}_{v}) \\ ={}&\text{R}(\boldsymbol{N}\boldsymbol{y};\mathbf{0})+(\boldsymbol{\beta}+\boldsymbol{\beta}_{u})'(\boldsymbol{N}\boldsymbol{X}- \boldsymbol{X}_{0})'(\boldsymbol{N}\boldsymbol{X}-\boldsymbol{X}_{0}) (\boldsymbol{\beta}+\boldsymbol{\beta}_{u}) \\ ={}&\text{R}(\boldsymbol{C}\boldsymbol{y};\mathbf{0})+(\boldsymbol{\beta}+\boldsymbol{\beta}_{u})'(\boldsymbol{C}\boldsymbol{X}- \boldsymbol{X}_{0})'(\boldsymbol{C}\boldsymbol{X}-\boldsymbol{X}_{0}) (\boldsymbol{\beta}+\boldsymbol{\beta}_{u}) \\ ={}&\text{R}(\boldsymbol{C}\boldsymbol{y};\boldsymbol{\beta}+\boldsymbol{\beta}_{u}), \end{aligned}$$

and hence $\text{(16)} \Leftrightarrow \text{R}(\boldsymbol{N}\boldsymbol{y};\boldsymbol{\beta}+\boldsymbol{\beta}_{v})=\text{R}(\boldsymbol{C}\boldsymbol{y};\boldsymbol{\beta}+\boldsymbol{\beta}_{u})\Leftrightarrow \text{R}(\boldsymbol{N}\boldsymbol{y}+\boldsymbol{P}_{v}\boldsymbol{v};\boldsymbol{\beta})=\text{R}(\boldsymbol{C}\boldsymbol{y}+\boldsymbol{P}_{u}\boldsymbol{u};\boldsymbol{\beta})$. Consequently, (14) means that, for any $\boldsymbol{\beta}\in\mathbb{R}^{p}$ and any predictor $\boldsymbol{N}\boldsymbol{y}+\boldsymbol{v}\in\mathscr{LN}$ of $\boldsymbol{y}_{0}$, if $\text{R}(\boldsymbol{N}\boldsymbol{y}+v;\boldsymbol{\beta}) \leq \text{R}(\boldsymbol{C}\boldsymbol{y}+\boldsymbol{u};\boldsymbol{\beta})$, then $\text{R}(\boldsymbol{N}\boldsymbol{y}+\boldsymbol{v};\boldsymbol{\beta})=\text{R}(\boldsymbol{C}\boldsymbol{y}+\boldsymbol{u};\boldsymbol{\beta})$, namely $\boldsymbol{C}\boldsymbol{y}+\boldsymbol{u} \sim \boldsymbol{\delta}$. □

### Remark 4.1

Theorem 4.1 shows the relation between the admissible homogeneous and nonhomogeneous linear predictors, and indicates that the admissibility of the homogeneous linear predictor is more significant. To derive an admissible predictor $\boldsymbol{C}\boldsymbol{y}+\boldsymbol {u}$ in $\mathscr{LN}$, we can derive the admissible predictor ***Cy*** in $\mathscr{LH}$ beforehand.

## Concluding remarks

In this paper, we investigate the admissibility of linear prediction in the generalized linear regression model under the quadratic loss function. Predictions are based on a composite target function that allows one to predict actual and average values of the unobserved regressand simultaneously, according to some practical needs. Necessary and sufficient conditions for the simultaneous prediction to be admissible are obtained in classes of homogeneous and nonhomogeneous linear predictors, respectively. Although the unbiased predictors of the composite target function are the unbiased predictors of the actual and average values of the unobserved regressand and vise versa, yet the admissibility of these predictors for each studied variables are different. Under some circumstances, the ridge predictor is admissible although it is biased. However, whether the admissible linear prediction is minimax under quadratic loss function is unclear. Further research on the minimaxity of admissible simultaneous prediction is in progress.

## Abbreviations

BLUP: Best linear unbiased predictor
MSE: Mean squared error
SPP: Simple projection predictor
BLUE: Best linear unbiased estimation
